# A Lead-In with Silibinin Prior to Triple-Therapy Translates into Favorable Treatment Outcomes in Difficult-To-Treat HIV/Hepatitis C Coinfected Patients

**DOI:** 10.1371/journal.pone.0133028

**Published:** 2015-07-15

**Authors:** Dominique L. Braun, Andri Rauch, Manel Aouri, Nina Durisch, Nadia Eberhard, Alexia Anagnostopoulos, Bruno Ledergerber, Beat Müllhaupt, Karin J. Metzner, Laurent Decosterd, Jürg Böni, Rainer Weber, Jan Fehr, Swiss HIV Cohort Study

**Affiliations:** 1 Division of Infectious Diseases and Hospital Epidemiology, University Hospital Zurich, University of Zurich, Zurich, Switzerland; 2 Institute of Medical Virology, University of Zurich, Zurich, Switzerland; 3 Department of Infectious Diseases, Bern University Hospital and University of Bern, Bern, Switzerland; 4 Division of Clinical Pharmacology, University Hospital Center, University of Lausanne, Lausanne, Switzerland; 5 Clinic for Gastroenterology and Hepatology, University Hospital Zurich, University of Zurich, Zurich, Switzerland; 6 Swiss National Center for Retroviruses, Institute of Medical Virology, University of Zurich, Zurich, Switzerland; Saint Louis University Division of Infectious Diseases and Immunology, UNITED STATES

## Abstract

**Background:**

The efficacy of first-generation protease inhibitor based triple-therapy against hepatitis C virus (HCV) infection is limited in HIV/HCV-coinfected patients with advanced liver fibrosis and non-response to previous peginterferon-ribavirin. These patients have a low chance of achieving a sustained virologic response (SVR) using first generation triple-therapy, with a success rate of only 20%. We investigated the efficacy and safety of lead-in therapy with intravenous silibinin followed by triple-therapy in this difficult-to-treat patient group.

**Methodology:**

Inclusion criteria were HIV/HCV coinfection with advanced liver fibrosis and documented previous treatment failure on peginterferon-ribavirin. The intervention was a lead-in therapy with intravenous silibinin 20 mg/kg/day for 14 days, followed by triple-therapy (peginterferon-ribavirin and telaprevir) for 12 weeks, and peginterferon-ribavirin alone for 36 weeks. Outcome measurements were HCV-RNA after silibinin lead-in and during triple-therapy, SVR data at week 12, and safety and tolerability of silibinin.

**Results:**

We examined sixteen HIV/HCV-coinfected patients with previous peginterferon-ribavirin failure, of whom 14 had a fibrosis grade METAVIR ≥F3. All were on successful antiretroviral therapy. Median (IQR) HCV-RNA decline after silibinin therapy was 2.65 (2.1–2.8) log10 copies/mL. Fifteen of sixteen patients (94%) had undetectable HCV RNA at weeks 4 and 12, eleven patients (69%) showed end-of-treatment response (i.e., undetectable HCV-RNA at week 48), and ten patients (63%) reached SVR at week 12 (SVR 12). Six of the sixteen patients (37%) did not reach SVR 12: One patient had rapid virologic response (RVR) (i.e., undetectable HCV-RNA at week 4) but stopped treatment at week 8 due to major depression. Five patients had RVR, but experienced viral breakthroughs at week 21, 22, 25, or 32, or a relapse at week 52. The HIV RNA remained below the limit of detection in all patients during the complete treatment period. No serious adverse events and no significant drug-drug interactions were associated with silibinin.

**Conclusion:**

A lead-in with silibinin before triple-therapy was safe and highly effective in difficult-to-treat HIV/HCV coinfected patients, with a pronounced HCV-RNA decline during the lead-in phase, which translates into 63% SVR. An add-on of intravenous silibinin to standard of care HCV treatment is worth further exploration in selected difficult-to-treat patients.

**Trial Registration:**

ClinicalTrials.gov NCT01816490

## Introduction

Treatment of hepatitis C virus (HCV) infection has currently entered a new era [1,2]. The availability of next-generation direct-acting antiviral drugs (DAAs) against HCV makes a cure possible for the majority of patients infected with HCV, almost regardless of HIV coinfection, fibrosis stage, previous response to standard of care therapy or genetic variations in the interleukin genotype [3–9]. However, using early DAAs, physicians were challenged by the limited efficacy and the high toxicity of first-generation HCV protease inhibitor based triple-therapy (i.e., telaprevir or boceprevir combined with peginterferon-ribavirin) [10], significant drug-drug interactions and the high pill burden which compromised patient adherence.

In 2011, we investigated as a proof of concept the effect of intravenous silibinin—an extract of the milk thistle *silybum marinum* and commercially available as Legalon SIL—on HCV-RNA decline and treatment outcome in HIV/HCV coinfected patients with advanced liver fibrosis/cirrhosis and previous failure of peginterferon-ribavirin therapy. These patients are most in need of immediate therapy as they are at highest risk for HCV-related morbidity and mortality [11]. We added intravenous silibinin as lead-in prior to triple-therapy in 16 HIV/HCV-coinfected patients with advanced liver fibrosis who had failed on previous peginterferon-ribavirin therapy. Encouraged by the excellent virologic responses of the first six pilot patients with a sustained virologic response (SVR) rate of 80% at week 24, we felt it important to share these data, published in June 2014 [12]. Here, we show the data about treatment outcomes and drug concentrations in all 16 patients.

## Methods

### Objectives

With this proof of concept study we aimed to assess the virologic outcomes of the lead-in with intravenous silibinin and the subsequent triple-therapy (i.e., telaprevir in combination with peginterferon-ribavirin), as well as the safety and tolerability of silibinin in HIV/HCV-coinfected patients with advanced liver fibrosis who failed on previous treatment with peginterferon-ribavirin.

### Study design and patient selection

**Fig 1:**
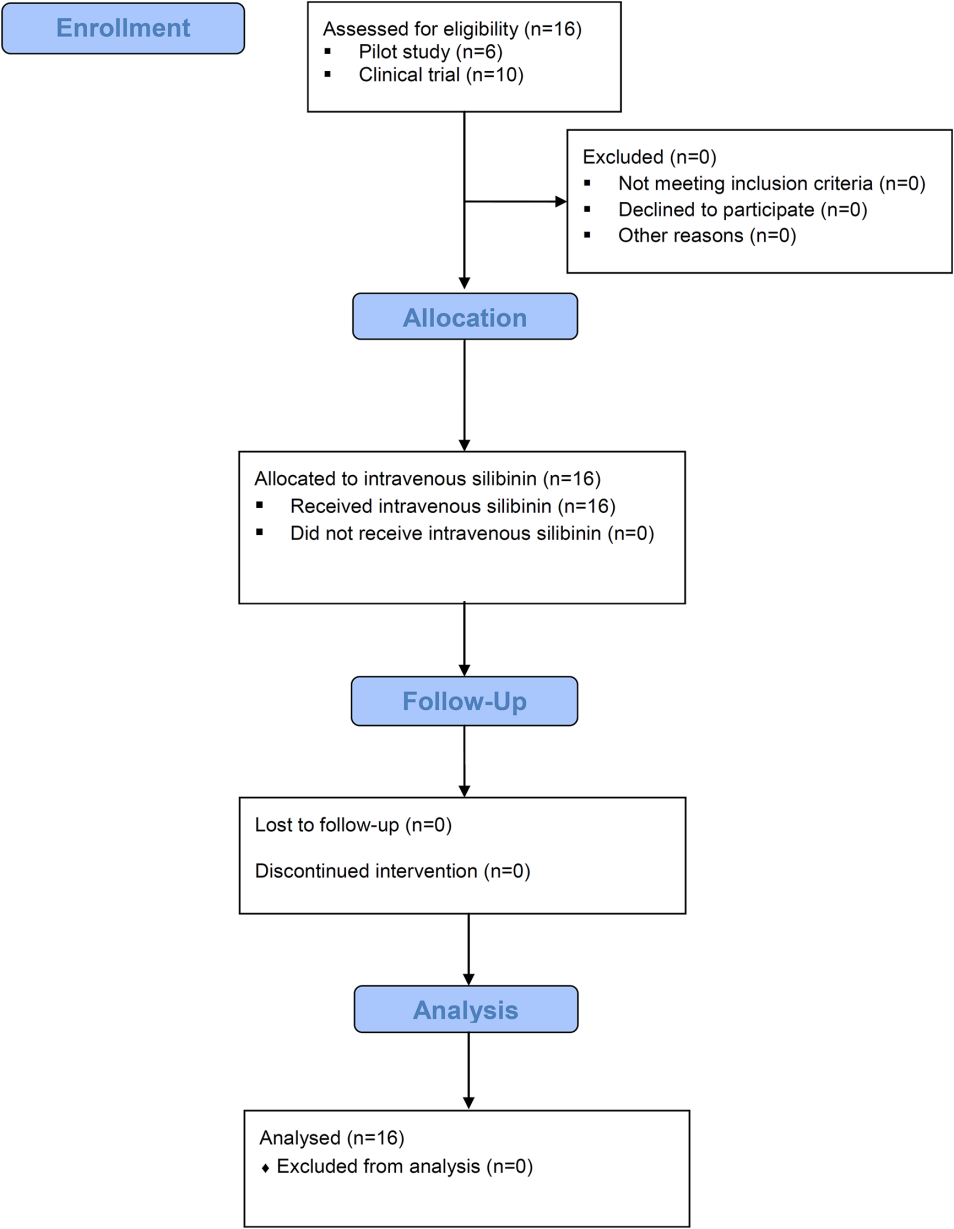
Flowchart of sixteen HIV/HCV coinfected patients allocated to a lead-in with intravenous silibinin.

Between May 2012 and October 2012 we prospectively enrolled six individuals coinfected with HIV/HCV genotype 1 within the pilot study called PRE-THISTLE. The outcome of these six patients has been previously published [12]. After that, between April 2013 and November 2013, we included another ten individuals coinfected with HIV/HCV genotype 1 in the THISTLE trial–the HIV/HCV silibinin trial, which is a phase II, multi-center, open-label, interventional study to evaluate the safety of intravenous silibinin and its effect on the hepatitis C virus load in treatment-experienced HIV/HCV coinfected individuals with advanced liver fibrosis in the Swiss HIV Cohort Study (SHCS) (http://clinicaltrials.gov, ID NCT01816490) (Fig 1 and data in S1 and S2 Files). The authors confirm that all ongoing and related trials for this drug/intervention are registered. Based on our inclusion and exclusion criteria we selected 16 patients within the SHCS who were eligible for the study. We did not evaluate further enrollment of patients since newer, better tolerable, and more effective DAAs became available at that time. The overall study duration was 15 days (i.e., the time-period including the administration of intravenous silibinin). Subsequent to THISTLE (i.e., at day 15) the study-subjects were included in SHCS trial #688. Trial #688 investigated the occurrence of HCV-resistance to the HCV protease inhibitors during triple-therapy. The duration of trial #688 was 48 weeks with a follow-up of 12 weeks (i.e., SVR 12 data). For the pilot (PRE-THISTLE) study, all six patients were enrolled at the University Hospital Zurich, University of Zurich. For the THSTLE trial, six patients were recruited at the University Hospital Zurich, University of Zurich, and four patients at the Bern University Hospital, University of Bern.

We treated all 16 patients (14 men and two women) with an intravenous silibinin lead-in and subsequently initiated triple-therapy. Patient selection was based on documented advanced liver fibrosis and a documented history of previous failure of standard-of-care peginterferon-ribavirin, including three different scenarios: (i) non-response (i.e., less than 2 log_10_ IU/mL decrease in HCV RNA from baseline to week 12 of therapy), (ii) partial response (i.e., more than 2 log_10_ IU/mL decrease in HCV RNA at week 12, but detectable HCV RNA at week 24) or (iii) viral breakthrough (i.e., undetectable HCV RNA at week 24, but detectable HCV RNA during treatment). Advanced liver fibrosis was defined as a METAVIR fibrosis score ≥2 in at least one documented liver biopsy since diagnosis of HCV infection. SVR at week 12 is defined as an undetectable HCV RNA level 12 weeks after the end of treatment. Of note, a SVR 12 correlates strongly with a permanent clearance of the virus and predicts a cure in 99 to 100% of patients. [13]

The ethical committee of the Canton Zurich approved the THISTLE trial on January 15^th^ 2013. A study amendement was approved by the ethical committee of the Canton Zurich on June 3^th^ 2013. Written informed consent was given by all study participants.

### Investigational product and mode of treatment

Intravenous silibinin has been commercially available as silibinin-C-2’, 3-dihydrogen succinate disodium salt (i.e., Legalon SIL) for 30 years for the treatment of hepatic intoxication by Amanita phalloides. In Austria, Legalon SIL was approved in December 2010 for treatment of chronically HCV-infected patients with non-response or partial response to peginterferon-ribavirin. In Switzerland, Legalon SIL is accepted for compassionate use for the same indication. All patients received lead-in therapy with intravenous silibinin at 20 mg/kg/day once daily for 14 days. Silibinin was administrated over one hour. After the 14-day intravenous silibinin lead-in treatment, patients started standard triple-therapy on day 15 (i.e. week 0), including telaprevir 375mg TID, pegylated interferon alfa-2a 180μg once a week, and weight-based daily ribavirin 1000mg (body weight <65kg) or 1200mg (body weight ≥65kg). Triple-therapy was continued for 12 weeks, followed by 36 weeks of peginterferon-ribavirin dual therapy.

### Assessment of HCV RNA, silibinin safety and tolerability

Laboratory assessments to ensure drug safety were made on days 1, 8, 12, and 15 of intravenous silibinin lead-in, and during subsequent triple therapy in weeks 1, 2, 4, 12, 24, 36, and 48, and additionally according to patient’s clinical state. These assessments included measurements of full blood count, electrolytes (sodium, potassium), aspartate aminotransferase, alanine aminotransferase, total and fractionated bilirubin, gamma-glutamyl transferase, total protein, alkaline phosphatase, creatinine, albumin, and international normalized ratio (INR). Serum HCV RNA levels were determined by the TaqMan PCR assay (Roche COBAS TaqMan v2.0, limit of detection 15 IU/mL). HCV RNA was measured on days 1, 8, and 15 of silibinin treatment. During triple-therapy, HCV viral load was monitored at weeks 2, 4, and 12. The tolerability of intravenous silibinin was determined by daily clinical assessment during the silibinin treatment period.

### Antiretroviral and silibinin drug levels

Plasma samples obtained from HIV-HCV infected individuals were isolated by immediate centrifugation at 1850 X g (3000 rpm) and stored at -80°C. For the plasma quantification of antiretroviral drugs, samples were inactivated for virus at 60°C for 60 minutes. Plasma levels were determined by liquid chromatography coupled with tandem mass spectrometry (LC-MS/MS) after protein precipitation with acetonitrile according to our previously reported validated analytical method [14]. The laboratory participates in an international external quality assurance program for antiretroviral drugs analysis (KKGT, Stichting Kwaliteitsbewaking Klinische Geneesmiddelanalyse en Toxicologie, Association for Quality Assessment in TDM and clinical Toxicology, The Hague, The Netherlands). Plasma trough levels for raltegravir (RAL), darunavir (DRV), atazanavir (ATV), and etravirine (ETV) were graphically extrapolated from measured concentrations, using population pharmacokinetic models previously published [15–17]. Reference values for efavirenz (EFV) were adopted from a study from Marzolini et al. [18]. The median trough concentrations for these agents in HIV-infected persons receiving the recommended dose are taken from the guidelines for the use of antiretroviral agents in HIV-1 infected adults and adolescents [19].

To measure silibinin plasma concentration, the plasma concentrations of silibinin isomers (A and B) and silibinin dihydrogen succinate isomers (A and B) were determined by LC/MS-MS. Of note, the range of plasma concentrations of the LC-MS/MS assay was selected according to the levels reported in the study from Sala et al [20]. Briefly, 100 μl of plasma was added to 20 μl of internal standard naringenin (IS) and extracted by protein precipitation. After evaporation and reconstitution, 20 μL was injected into a Select- HSS T3 (2.1x75 mm, 3.5 μm) (Waters, Milford, MA, USA).

Chromatographic separations were performed using a gradient program with 10 mM ammonium formate containing 0.1% formic acid and acetonitrile with 0.1% formic acid. Analytes were quantified by electrospray ionization-triple quadrupole mass spectrometry using the selected reaction monitoring detection in the negative mode. Importantly, the selected gradient program allows adequate separation to quantify isomers A and B individually. The method has a mean inter-day coefficient of variation (CV) % of <10% and an inter-day deviation from nominal values of <7%.

### Statistical analysis

We used an intention-to-continue treatment approach to calculate virologic responses as percentage of patients with undetectable HCV-RNA, using all patients who started treatment as denominator. Plasma concentrations are presented as median (range). Two-tailed paired t-tests were conducted to detect difference in trough levels of antiretroviral drugs between pre- and post-administration of silibinin. GraphPad Prism 6 (GraphPad Software, La Jolla, CA) was used with an alpha of 0.05.

## Results

### Patient characteristics

The 16 patients had a median age of 50 years (range 38–60) (Table 1). The most prevalent HCV transmission mode was via intravenous drug use (n = 12), followed by sexual transmission (n = 2), contaminated blood products (n = 1), and unknown (n = 1). All patients were on fully suppressive antiretroviral therapy (ART) (i.e., <20 HIV-RNA copies/mL plasma), and had a median CD4^+^ T-cell count of 586 cells/μL (range 175–2529). Fourteen patients (88%) were infected with HCV genotype 1a, and one each with genotypes 1b and 1e. Nine patients (57%) had a METAVIR fibrosis score F4, five (31%) F3, and two (12%) F2. Previous peginterferon-ribavirin therapy in the 16 patients elicited 11 non-responses, 4 partial responses, and one viral breakthrough. The ART regimens of the patients included ATV, DRV, RAL, EFV, tenofovir (TDF), emtricitabine (FTC), abacavir (ABC), and lamivudine (3TC).

**Table 1:** Baseline characteristics of sixteen HIV/HCV coinfected patients.

Patient	Age	Sex	CD4+	ART	GT	METAVIR	Fibroscan	Preceding Tx
	years		cells/μL				kPa	
**1**	49	m	396	RAL,ATV/r,TDF	1a	F3	35.8	NR
**2**	48	f	520	RAL,TDF/FTC	1e	F3	19.8	NR
**3**	50	m	627	RAL,ATV/r,TDF	1a	F3	18.4	Breakthrough
**4**	47	m	686	RAL,ABC/3TC	1b	F3	NA	PR
**5**	38	m	175	RAL,EFV,TDF	1a	F3	24.6	NR
**6**	50	m	504	ATV/r,RAL,TDF/FTC	1a	F4	17.3	PR
**7**	45	m	433	RAL,TDF/FTC	1a	F4	12	NR
**8**	53	m	726	ATV/r,TDF/FTC	1a	F4	43.1	PR
**9**	44	m	501	ATV/r,RAL,TDF/FTC	1a	F4	14.4	NR
**10**	48	m	430	ATV/r,RAL,TDF	1a	F4	37.4	NR
**11**	56	m	378	ETR,TDF/FTC	1a	F2	6.3	PR
**12**	52	m	248	RAL,TDF/FTC	1a	F4	17.3	NR
**13**	60	m	744	DRV/r,TDF/FTC	1a	F2	5.8	NR
**14**	43	m	2529	RAL,TDF/FTC	1a	F4	13.8	NR
**15**	51	f	586	RAL,TDF/FTC	1a	F4	8.8	NR
**16**	55	m	1198	DRV/r,TDF/FTC,ETR	1a	F4	6.8	NR

Abbreviations: ART: antiretroviral therapy; GT: genotype; Tx: therapyNR: null response; PR: partial responseRAL: raltegravir; ATV/r: atazanavir/ritonavir; EFV: efavirenz; TDF: tenofovir; FTC: emtricitabine; 3TC: lamivudine; ABC: abacavir; DRV/r: darunavir/ritonavir; ETR: etravirine

### Drug levels of Silibinin and concurrent antiretroviral therapy

Plasma concentrations for silibinin A, B isomers, silibinin dihydrogen succinate A, B isomers and antiretroviral drug levels were measured at several occasions at days 1 and 12 for C_max_ and days 2, 4, 8, 12, and 15 for C_min_. Median (range) trough levels for silibinin A, B isomers and silibinin dihydrogen succinate A, B isomers are reported in Table 2. Of note, the C_min_ of isomer A of both silibinin dihydrogen succinate and silibinin were 2- and 7-fold higher than the corresponding values of isomer B [20].

**Table 2:** Median Plasma concentrations (range) of silibinin, dihydrogen succinate silibinin and their respective isomers A and B.

	Median Cmax (Range) (μg/mL)	Median Cmin (range) (μg/mL)
**Silibinin A**	0.62 (0.29–1.07)	0.09 (0–0.48)
**Silibinin B**	0.25 (0.08–0.78)	0.012 (0–0.22)
**Dihydrosuccinate silibinin A**	81.54 (24.94–7602)	1.03 (0–18.59)
**Dihydrosuccinate silibinin B**	71.31 (11.15–1971)	0.097 (0–16.15)

**Fig 2:**
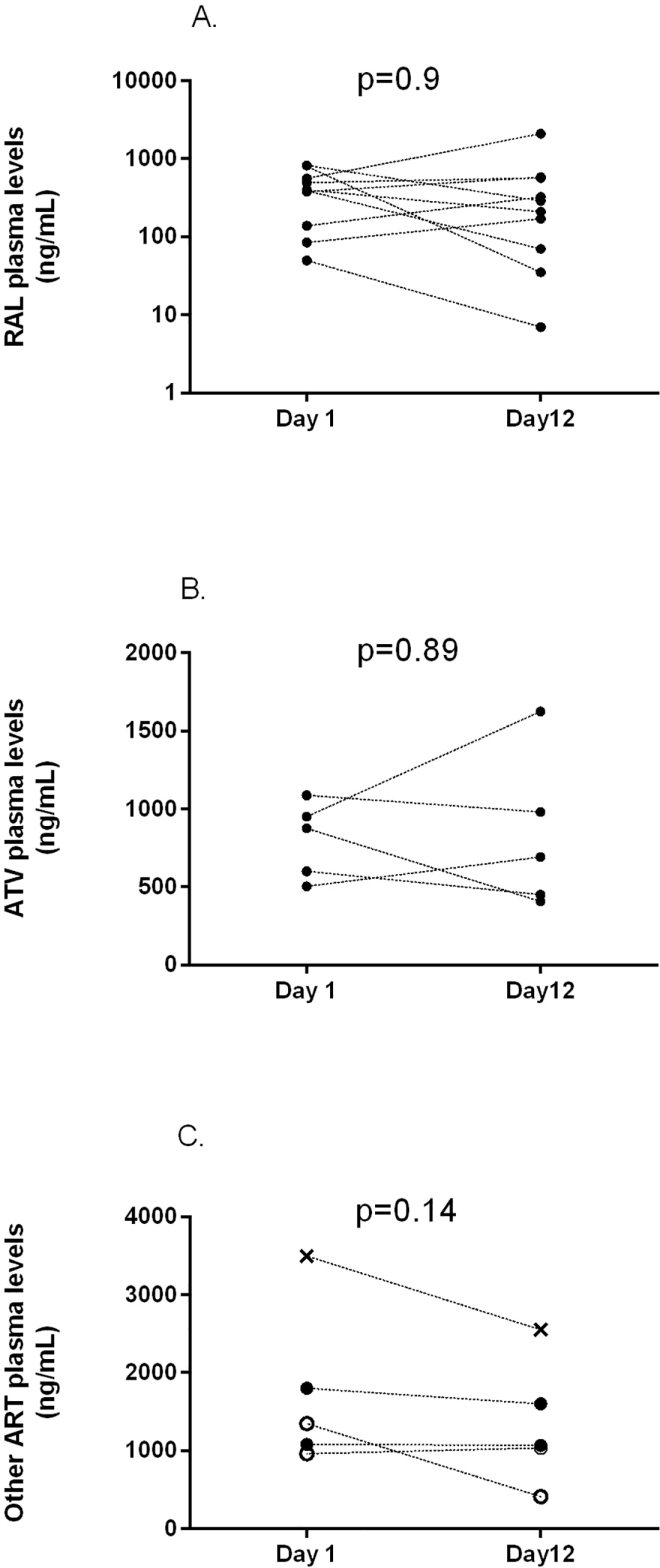
Plasma trough concentrations in patients before (day 1) and during (day 12) silibinin treatment. **A** Raltegravir levels (n = 10) **B** Atazanavir plasma levels (n = 5) **C x**—**-x** Efavirenz (n = 1) ●—- ● Darunavir **(**n = 2) ○—-○Etravirine (n = 2).

Most patients received RAL (n = 13) in combination with ATV/r (n = 5), EFV (n = 1), or DRV/r (n = 1). ETV was prescribed in 2 patients, combined with DRV/r in one participant. Among the 16 patients who completed the study, ART plasma levels are reported for 15 patients. One patient was excluded, because there was no plasma sample for drug level measurement available. As depicted in Fig 2, trough levels of the antiretroviral drugs ATV/r and RAL were not influenced by silibinin coadministration (p = 0.89, p = 0.90, respectively). Similarly, ETV, EFV, and DRV/r plasma levels were not influenced by silibinin administration (p = 0.14).

### HCV and HIV RNA during silibinin lead-in and HCV triple therapy

**Fig 3:**
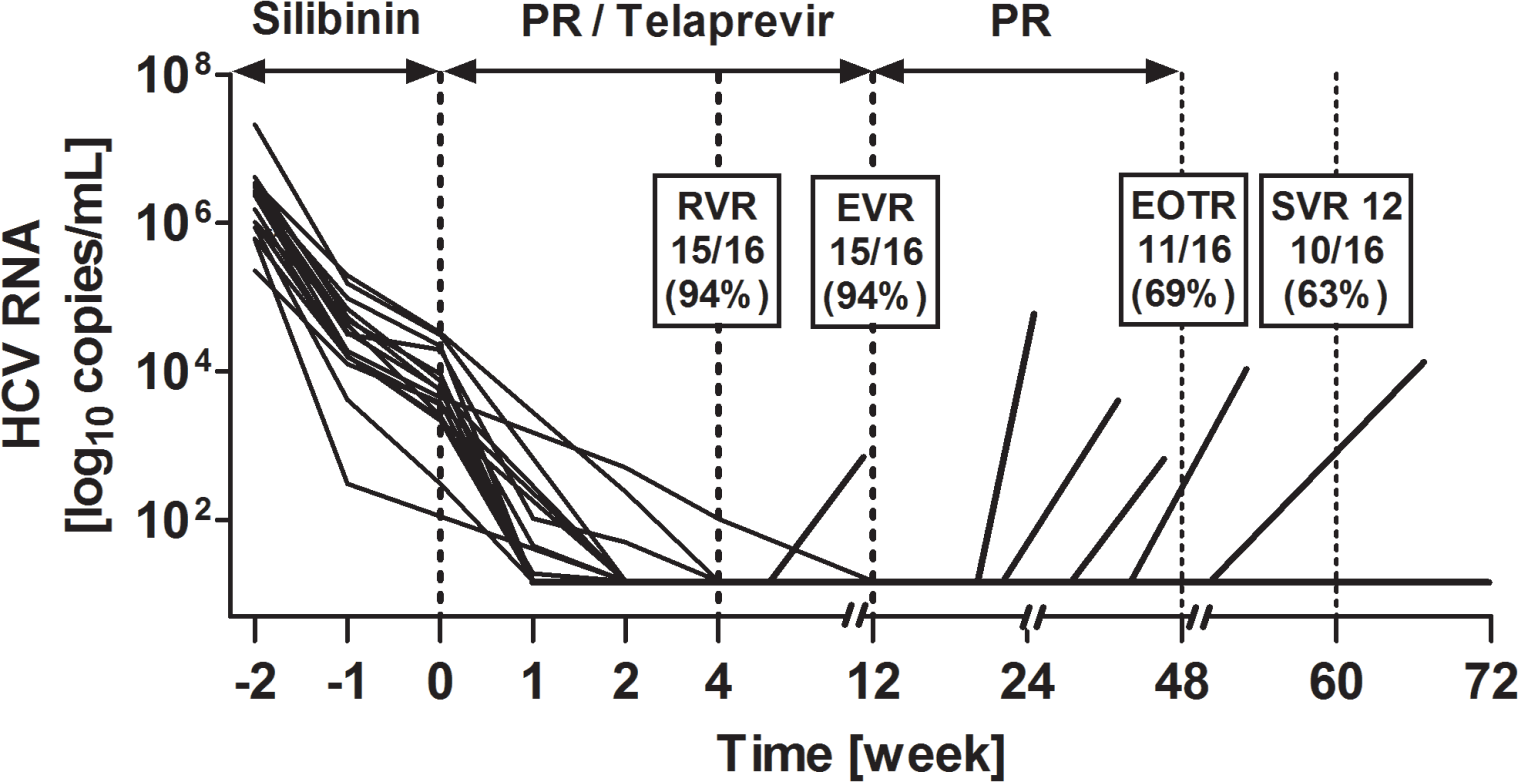
HCV-RNA viral load in sixteen patients during lead-in therapy with intravenous silibinin treatment for 2 weeks followed by triple-therapy for 12 weeks. One patient discontinued treatment due to major depression before week 8. Five patients experienced viral breakthroughs at weeks 21, 22, 28, 32, or 52.RVR: rapid virologic response; EVR: early virologic response; EOTR: end of treatment response; SVR: sustained virologic response; PR: pegylated interferon and ribavirin.

During the 14 days of intravenous silibinin therapy, HCV RNA declined by >5 log_10_ IU/mL (n = 1), 3–5 (n = 2), 2–3 log_10_ IU/mL (n = 12), and 1–2 log_10_ IU/mL (n = 1); the median interquartile range (IQR) of HCV RNA decline was 2.65 (2.1–2.8) log_10_ IU/mL (Fig 3). After the silibinin lead-in therapy and initiation of triple therapy, 13 of 16 patients (81%) reached a HCV RNA below the limit of detection at week 2 of triple-therapy, 15 of 16 patients (94%) at week 4 of triple therapy, and 15 of 16 patients (94%) had undetectable HCV RNA at week 12 of triple therapy. Eleven of sixteen patients (69%) had an end-of-treatment-response (i.e., undetectable HCV RNA at week 48), and 10 of 16 patients (63%) showed a SVR 12 thereafter (Fig 3).

Of all sixteen study-participants, six patients (37%) did not reach SVR 12: One patient had a rapid virologic response (RVR) but stopped treatment at week 8 due to major depression. Five patients had a RVR, but experienced a viral breakthroughs at week 21, 22, 25, or 32, or a relapse at week 52. The HIV RNA remained suppressed in all patients during the complete treatment period, and at SVR 12.

### Adverse events and laboratory abnormalities during silibinin treatment

Intravenous silibinin treatment was well tolerated in all patients without any serious adverse events (Table 3). Mild or moderate adverse events occurred in 14 of 16 patients (88%), including sensations of heat (n = 7), headaches (n = 3), nausea (n = 1), diarrhoea (n = 1), and mild exanthema (n = 1). The most frequent laboratory abnormality observed was an increase in bilirubin in 11 of 16 patients (69%) with a peak level of 78μmol/L (normal range: <21), however, this increase was not clinically significant and resolved spontaneously after the end of silibinin treatment. No abnormalities in full blood count, transaminases or kidney function occurred during silibinin treatment.

**Table 3:** Adverse events and laboratory abnormalities during silibinin treatment.

Event	Number of patients (%)n = 16
Discontinuation because of an adverse event	0
Any serious adverse event	0
Any adverse event	14 (88)
Adverse event probably or definitely related to silibinin^1^	13 (81)
Sensation of heat during silibinin infusion	7 (44)
Headache	3 (19)
Nausea	1 (6)
Diarrhoea	1 (6)
Mild exanthema	1 (6)
Laboratory abnormalities	
>1.5x increase in bilirubin^2^	11 (69)

^1^ All adverse events were of mild or moderate intensity and resolved spontaneously

^2^ The Increase in bilirubin was clinically not significant and resolved spontaneously

## Discussion

We demonstrated as a proof of concept that an intravenous silibinin lead-in was safe, well tolerated and highly effective in a difficult-to-treat patient population and resulted in a pronounced HCV RNA decline during the lead-in phase. The subsequent initiation of telaprevir-based triple-therapy led to a SVR 12 in ten of sixteen (63%) HIV/HCV-coinfected patients with advanced liver fibrosis/cirrhosis and previous failure of peginterferon-ribavirin.

We recently published data on the first six patients treated with a silibinin lead-in followed by triple-therapy and reported an SVR rate of 80% [12]. Meanwhile ten additional patients were treated with a silibinin lead-in. One patient discontinued treatment due to side effects and another four patients experienced viral breakthrough. Thus, the SVR rate decreased to 63%. However, a SVR 12 of 63% is still remarkably high compared with reported treatment outcomes in similar patient groups. Data from the CUPIC trial revealed that only 20% of the patients with cirrhosis who did not respond to previous peginterferon-ribavirin treatment achieved an SVR with triple therapy [21]. The REALIZE trial reported on-treatment virologic failure in up to 50% of patients [22]. Virologic failure could be driven by the development of HCV protease inhibitor resistance and the insensitivity of the hepatic cells to peginterferon-ribavirin [23]. We hypothesize that the higher SVR rate in our patient group is mainly related to the lead-in treatment with silibinin. Silibinin led to a rapid HCV RNA decline and presumably prevented the development of resistance against telaprevir. An additional explanation might be that treatment discontinuations due to triple-therapy related side effects were very rare in our cohort and occurred in only one patient (6%). A European cohort including difficult-to-treat coinfected patients recently reported treatment discontinuations in 5 of 21 patients (23%) due to side effects [24].

We acknowledge that in the light of the extremely potent and well tolerated next-generation DAAs the current value of silibinin for treatment of HCV infection is limited [25]. However, as a proof of concept we demonstrated that a silibinin lead-in was very effective and very well tolerated in difficult-to-treat HIV/HCV coinfected patients and caused no significant drug-drug interactions. To the best of our knowledge, the plasma concentrations of silibinin isomers and prodrugs (silibinin dihydrogen succinate A and B) are reported for the first time in HIV/HCV coinfected patients. These levels provide information about the range of C_max_ and C_min_ observed in this population and indicate the wide variability between individuals. The finding that silibinin does not influence the pharmacokinetics of antiretroviral drugs is in line with a previous report from Molto et al. [26].

We would like to share our data since a silibinin add-on could have some benefit in special clinical settings for selected patients: First, in HIV/HCV coinfected patients on salvage ART in whom significant drug-drug interactions may limit the use of some newer DAAs. For example, HIV protease inhibitors cannot be administrated together with the HCV protease inhibitor simeprevir or with the new compounds MK-5172 and MK 8742 [6, 25, 27]. Second, to improve treatment success with DAAs and/or prevent development of resistance to DAAs with a low resistance barrier [28]. Third, in patients not tolerating newer DAAs but in clinical need for immediate treatment. However, further studies should be done including these selected populations to determine how best to use silibinin to improve outcomes for these difficult-to-treat patients.

Apart from its anti-HCV effect, silibinin shows a broad-spectrum antiviral activity *in vitro* and therefore might serve as an investigational antiviral agent in the setting of life-threatening, to date untreatable viral diseases (e.g., severe respiratory syncytial virus infections in immunocompromised patients) [29, 30]. In addition, silibinin is reported to have potential as a hepatitis B/hepatitis delta entry inhibitor through down-regulation of virus specific receptors and effects on envelope proteins [31], and as therapeutic agent for treatment of non-alcoholic fatty liver disease and alcoholic liver cirrhosis [32, 33]. The possible uses of silibinin may become even more interesting once this drug becomes available as oral regimen with the ability to achieve high serum concentrations.

In conclusion, a lead-in course with intravenous silibinin prior to the initiation of triple-therapy resulted in a 63% SVR 12 rate in ten of sixteen HIV/HCV coinfected patients with advanced liver fibrosis and previous failure to peginterferon-ribavirin. Tolerability and safety of silibinin was favorable and no significant drug-drug interactions with ART occurred. Due to its anti-viral and anti-inflammatory properties, silibinin may serve in the future as part of the cure for hepatitis C and other viral infections and liver diseases.

## Supporting Information

S1 CONSORT Checklist CONSORT Checklist. (DOC)

S1 Protocol Clinical study protocol THISTLE–The HIV-HCV Silibinin Trial. A phase II, multi-center, open-label, interventional study to evaluate the safety of intravenous silibinin and its effect on the hepatitis C virus load in treatment-experienced HCV-HIV co-infected individuals with advanced liver fibrosis in the Swiss HIV Cohort Study (SHCS).
(PDF)
