# Correction: A Lead-In with Silibinin Prior to Triple-Therapy Translates into Favorable Treatment Outcomes in Difficult-To-Treat HIV/Hepatitis C Coinfected Patients

**DOI:** 10.1371/journal.pone.0135547

**Published:** 2015-08-07

**Authors:** The PLOS ONE Staff

There are errors in the Funding and Competing interests sections. In the Funding statement, the membership of the Swiss HIV Cohort Study should not have been included, and some information in the originally published Competing interests statement, along with funding from Max Zeller Söhne AG, should have been included. The correct Funding information is as follows, with the additions in bold:

**This study was funded by commercial companies, the Rottapharm|Madaus Group and Max Zeller Söhne AG. DLB received a grant from Rottapharm|Madaus and was supported by the University of Zurich’s Clinical research Priority Program (CRPP) “Viral infectious diseases: Zurich Primary HIV Infection Study” and by the matching fund program of the University Hospital of Zurich.** This study has been conducted within the framework of the Swiss HIV Cohort Study (SHCS), which is funded by the Swiss National Science Foundation (grant number 33CS30_134277, SHCS project 688). **AR declared remuneration for patient recruitment and received money from Janssen Cilag for serving on their advisory board. BL received money from Gilead for consultancy and from Gilead and GSK as payment for lectures. JB received money from the Federal Committee for Sexual Health, Switzerland. JF received a grant from Rottapharm|Madaus and is a member of the advisory board of Merck Sharp & Dome and Janssen and received unrestricted and travel grants from Gilead, Merck Sharp & Dome, Janssen, Bristol-Myers Squibb, Roche, VIIV, Abbott, and Boehringer Ingelheim. KJM has received travel grants and honoraria from Gilead Sciences, Roche Diagnostics, Tibotec, Bristol-Myers Squibb, Merck Sharp & Dohme, and Abbott; the University of Zurich has received research grants from Gilead Sciences, Roche Diagnostics, and Merck Sharp & Dohme for studies on which KJM serves as principal investigator and advisory board honoraria from Gilead Sciences and ViiV. RW received travel grants from Abbott, Boehringer Ingelheim, Bristol-Myers Squibb, Gilead Sciences, GlaxoSmithKline, Merck Sharp & Dome, Pfizer, Roche, TRB Chemedica and Tibotec. BM has received research grants from Roche and Gilead.** The funders had no role in study design, data collection and analysis, decision to publish, or preparation of the manuscript.

In the Competing interests statement, the provider of the investigational medicine product should have been included. The correct Competing interests statement is as follows, with the addition in bold:

The authors have read the journal's policy and the authors of this manuscript have the following competing interests: **The investigational medicine product was provided by the Rottapharm|Madaus Group.** DLB received a grant from Rottapharm|Madaus. AR declared remuneration for patient recruitment and received money from Janssen Cilag for serving on their advisory board. BL received money from Gilead for consultancy and from Gilead and GSK as payment for lectures. JB received money from the Federal Committee for Sexual Health, Switzerland. JF received a grant from Rottapharm|Madaus and is a member of the advisory board of Merck Sharp & Dome and Janssen and received unrestricted and travel grants from Gilead, Merck Sharp & Dome, Janssen, Bristol-Myers Squibb, Roche, VIIV, Abbott, and Boehringer Ingelheim. KJM has received travel grants and honoraria from Gilead Sciences, Roche Diagnostics, Tibotec, Bristol-Myers Squibb, Merck Sharp & Dohme, and Abbott; the University of Zurich has received research grants from Gilead Sciences, Roche Diagnostics, and Merck Sharp & Dohme for studies on which KJM serves as principal investigator and advisory board honoraria from Gilead Sciences and ViiV. RW received travel grants from Abbott, Boehringer Ingelheim, Bristol-Myers Squibb, Gilead Sciences, GlaxoSmithKline, Merck Sharp & Dome, Pfizer, Roche, TRB Chemedica and Tibotec. BM has served as an advisory board member for Roche, MSD, Janssen Therapeutics, AbbVie, Boehringer Ingelheim, Gilead and BMS; as a consultant for Gilead and AbbVie; and has received research grants from Roche and Gilead. This does not alter our adherence to PLOS ONE policies on sharing data and materials.
