# Supplementary material for: A Lead-In with Silibinin Prior to Triple-Therapy Translates into Favorable Treatment Outcomes in Difficult-To-Treat HIV/Hepatitis C Coinfected Patients
Source: PLoS One. 2015 Jul 15;10(7):e0133028. doi: 10.1371/journal.pone.0133028 (PMC4503454; doi:10.1371/journal.pone.0133028)
Supplement: S1 Protocol — A phase II, multi-center, open-label, interventional study to evaluate the safety of intravenous silibinin and its effect on the hepatitis C virus load in treatment-experienced HCV-HIV co-infected individuals with advanced liver fibrosis in the Swiss HIV Cohort Study (SHCS). (PDF) [file pone.0133028.s002.pdf]

Clinical Study Protocol

A phase II, multi-center, open-label, interventional study to evaluate the safety of intravenous silibinin (iSIL) and its effect on the hepatitis C virus load in treatment-experienced HCV-HIV co-infected individuals with advanced liver fibrosis in the Swiss HIV Cohort Study (SHCS)

THISTLE – The HIV-HCV Silibinin Trial

Sponsor: Jan Sven Fehr, MD

Attending physician

Divison of Infectious Diseases and Hospital Epidemiology

University Hospital Zurich

University of Zurich

Rämistrasse 100

8097 Zürich

E-Mail: jan.fehr@usz.ch

Phone: +41 44 255 34 02

Study Product: Intravenous Silibinin (iSIL), marketed as Legalon ® SIL

Protocol Version and Date: Version number 4, validity date May 16th 2013

CONFIDENTIAL

The information contained in this document is confidential and the property of the division for infectious diseases and hospital epidemiology of the University Hospital Zurich. The information may not - in full or in part - be transmitted, reproduced, published, or disclosed to others than the applicable Independent Ethics Committee(s) and Regulatory Authority(ies) without prior written authorization from division for infectious diseases and hospital epidemiology of the University Hospital Zurich except to the extent necessary to obtain informed consent from those subjects who will participate in the study.

STUDY SYNOPSIS

**Sponsor**	Jan Fehr, MD
**Study Title:**	A phase II, multi-center, open-label, interventional study to evaluate the safety of intravenous silibinin (iSIL) and its effect on the hepatitis C virus load in treatment-experienced HCV-HIV co-infected individuals with advanced liver fibrosis in the Swiss HIV Cohort Study (SHCS).
**Short Title/Study ID:**	THISTLE – The HIV-HCV Silibinin Trial
**Protocol Version and Date:**	Version number 4, validity date May 16th 2013.
**Clinical Phase:**	Phase 2
**Methodology:**	Multi-center, open-label, interventional study.
**Study Duration:**	Screening: 14 days<br>Treatment: 14 days (day 1 to 14)<br>Follow-up: 1 day (day 15)
**Study Center(s):**	Multi-centre with seven centers involved (Basel, Berne, St. Gallen, Lausanne, Geneva, Lugano and Zurich).
**Principal Investigators:**	Dominique Braun, Principal Investigator Zurich<br>University Hospital Zurich<br><br>Andri Rauch, Principal Investigator Berne<br>University Hospital Berne<br><br>Marcel Stöckle, Principal Investigator Basel<br>University Hospital Basel<br><br>Patrick Schmid, Principal Investigator Sankt Gallen<br>Cantonal Hospital Sankt Gallen<br><br>Juan Ambrosioni, Principal Investigator Geneva<br>University Hospital Geneva<br><br>Matthias Cavassini, Principal Investigator Lausanne<br>University Hospital Lausanne<br><br>Enos Bernasconi, Principal Investigator Lugano<br>Regional Hospital Lugano

**Objective(s)/ Outcome(s):**	The aim of this study is to evaluate the safety of a 14 days lead-in course with Legalon® SIL (iSIL) and its effect on the hepatitis C virus load in HCV-HIV co-infected individuals who failed previous treatment with PR.<br><br>The specific aims are:<br><br>Primary aims:<br>• To assess the safety of iSIL in the context of HCV-HIV co-infected patients and antiretroviral treatment (ART)<br>• To evaluate the kinetics of the decline in HCV RNA from baseline to the end of a 2 weeks treatment with iSIL.<br><br>Secondary aims:<br>• To assess the efficacy of iSIL in a variety of clinically meaningful virological responses<br>• To evaluate drug levels of iSIL and to assess its influence on the drug-levels of co-administrated ART.<br><br>Primary endpoint:<br>• Frequency of adverse events during iSIL treatment.<br>• Kinetics of the decline in HCV-RNA after 2 weeks of iSIL treatment<br><br>Secondary endpoint:<br>• Drug levels of iSIL and its influence on the drug-level of co-administrated ART.<br>• Proportion of patients with HIV virological failure, i.e. confirmed viremia >50cp/ml.
**Number of Subjects:**	A minimum of 20 subjects (Basel: 3, Berne: 4, Geneva: 3, Lausanne: 2, Lugano: 1, St. Gallen: 2, Zurich 5)
**Diagnosis and Main Inclusion Criteria:**	HCV-HIV co-infected SHCS participants with chronic HCV-genotype 1 infection and documented previous null-response (NR) or partial-response (paR) to standard therapy with SOC and in urgent need of treatment (i.e. METAVIR fibrosis score ≥ 2 or ISHAK fibrosis score ≥4 or fibroscan stiffness ≥ 9.5kPa) will be included.<br><br>Inclusion criteria<br>• ≥ 18 years<br>• HIV-HCV co-infection<br>• HCV Genotype 1 infection<br>• At least one liver biopsy since diagnosis of HCV-infection<br>• Fibrosis score METAVIR ≥ 2 documented by biopsy OR a stiffness ≥ 7.0kPa documented by fibroscan during the previous 12 months.<br>• Documented previous null-response or partial-response to SOC

**Main Exclusion Criteria:**	Exclusion criteria:<br>• Contraindications to the study drug under study, e.g. known hypersensitivity or allergy to any ingredient of the study drug<br>• Patients in need of ART with HIV virological failure (≥ 400 copies/ml) in the last 3 months
**Study Product, Dose, Route, Regimen:**	Intravenous Silibinin (iSIL) marketed in its esterified modification silibinin-C-2, 3-dihydrogen succinate and disodium salt as Legalon® SIL. All included patients will receive a lead-in therapy with iSIL in a dosage of 20mg/kg/day intravenously for 14 days once a day (day 1 to day 14). All study-subjects are treated outpatient.<br><br>On the end of the THISTLE study (day 15, i.e the day following the last iSIL administration) the subjects will be subsequently treated with standard of care triple therapy with telaprevir plus pegylated interferon and ribavirin (PR).
**Duration of administration:**	iSIL is administrated intravenously during 1-4 hours: at day one during 4 hours and then, if tolerated, from the second day during 2 hours and then, if tolerated from the third day during 1 hour in a dosage of 20mg/kg once daily for 14 days.
**Reference therapy, Dose, Route, Regimen:**	Not applicable due to the study design.
**Study Schedule:**	First patient enrolled: April 2013<br>Last patient enrolled: December 2013
**Statistical Methodology:**	The analyses for this non-comparative study will be purely descriptive. The decline of HCV RNA during the lead-in phase (day +1 to day +15) will be expressed in log10 IU/ml using methods for left-censored data in case of undetectable values are encountered. We will also attempt to analyse factors associated with HCV RNA response in mixed effects models taking into account all measured HCV values from day -14 to day 15. Adverse events will be reported in percentages (with 95% CI) of patients experiencing an event (for clinical events) or the percentages below/above 1x and 2x normal range limits for laboratory values.
**GCP Statement:**	This study will be conducted in compliance with the protocol, the current version of the Declaration of Helsinki, and ICH-GCP as well as all national legal and regulatory requirements. The study will also follow the respective SAMW Guideline regarding the collection of human biological material (Biobanking).

STUDY SCHEDULE

Study Periods	***Screen-ing***	***Treatment Period***														
Visit	***1***	***2***	***3***	***4***	***5***	***6***	***7***	***8***	***9***	***10***	***11***	***12***	***13***	***14***	***15***	***16***
Day	***-14 ± 7d***	***+1***	***2***	***3***	***4***	***5***	***6***	***7***	***8***	***9***	***10***	***11***	***12***	***13***	***14***	***15***
Subject Information and Informed Consent	*x*															
Demographics	*x*															
Medical History	*x*															
In- /Exclusion Criteria	*x*	*x*														
Physical Examination	*x*	*x*	*x*		*x*				*x*				*x*			*x*
Vital Signs	*x*	*x*	*x*	*x*	*x*	*x*	*x*	*x*	*x*	*x*	*x*	*x*	*x*	*x*	*x*	*x*
Safety laboratory	*x*	*x*	*x*		*x*				*x*				*x*			*x*
HIV-RNA	*x*	*x*														*x*
HCV-RNA	*x*	*x*	*x*	*x*	*x*	*x*	*x*	*x*	*x*	*x*	*x*	*x*	*x*	*x*	*x*	*x*
CD3/4/8 cells		*x*														*x*
Interleukin 28B	*x*															
Pregnancy Test	*x*															*x*
Plasma aliquot	*x*	*x*							*x*				*x*			*x*
PBMC[1]		*x*							*x*				*x*			*x*
iSIL drug level		*x*	*x*[2]		*x*[2]				*x*[2]				*x*			*x*
ART drug level		*x*											*x*			
Administer Study Medication		*x*	*x*	*x*	*x*	*x*	*x*	*x*	*x*	*x*	*x*	*x*	*x*	*x*	*x*	
Concomitant Therapy	*x*	*x*	*x*	*x*	*x*	*x*	*x*	*x*	*x*	*x*	*x*	*x*	*x*	*x*	*x*	*x*
Adverse Events		*x*	*x*	*x*	*x*	*x*	*x*	*x*	*x*	*x*	*x*	*x*	*x*	*x*	*x*	*x*

[1] *to be done only at Zurich site*
[2] *to be done only at Zurich and Lausanne site*

TABLE OF CONTENTS

1 LIST OF ABBREVIATIONS AND DEFINITIONS OF TERMS 9
2 INVESTIGATORS AND STUDY ADMINISTRATIVE STRUCTURE 10
3 ETHICS 14
3.1 Independent Ethics Committee (IEC) and Regulatory Authorities 14
3.2 Ethical Conduct of the Study 14
3.3 Subject Information and Informed Consent 14
4 INTRODUCTION 15
4.1 Background and Rationale 15
4.2 Investigational Product 16
4.3 Preclinical Data 17
4.4 Clinical Data to Date 17
4.5 Dose Rationale 18
4.6 Risk/Benefits and Ethical Considerations 18
5 STUDY OBJECTIVES AND OUTCOMES 20
5.1 Primary Variable(s) 20
5.2 Secondary Variables 20
6 STUDY DESIGN AND COURSE OF STUDY 21
6.1 General Design 21
6.2 Study visits 22
7 SUBJECT SELECTION AND WITHDRAWAL 23
7.1 Inclusion Criteria 23
7.2 Exclusion Criteria 23
7.3 Subject Recruitment and Screening 24
7.4 Early Withdrawal of Subjects 25
7.5 Early Termination of Study 26
8 TREATMENTS 27
8.1 Treatments to be administered 27
8.2 Identity of Investigational Product(s) 27
8.3 Method of Assigning Subjects to Treatment Groups 28
8.4 Treatment Compliance 28
8.5 Prior and Concomitant Therapy 28
8.6 Packaging, Labelling and Supply 28
8.7 Blinding 29
8.8 Storage Conditions 29
8.9 Study Drug Accountability 29
8.10 Return or Destruction of Study Drug 30
9 STUDY PROCEDURES 31
9.1 Day – 14 (Screening visit) 31
9.2 Day + 1 (baseline visit), immediately previous to iSIL administration 31
9.3 Day 2 (visit 3) 33
9.4 Day 3 (visit 4) 34

9.5 Day 4 (visit 5) ... 34
9.6 Day 5 (visit 6) ... 35
9.7 Day 6 (visit 7) ... 36
9.8 Day 7 (visit 8) ... 36
9.9 Day 8 (visit 9) ... 36
9.10 Day 9 (visit 10) ... 37
9.11 Day 10 (visit 11) ... 38
9.12 Day 11 (visit 12) ... 38
9.13 Day 12 (visit 13) ... 38
9.14 Day 13 (visit 14) ... 40
9.15 Day 14 (visit 15) ... 40
9.16 Day 15 (visit 16, end of THISTLE) ... 41
9.17 Sample preparation, storage, and shipment ... 42
10 SAFETY ... 45
10.1 Definition of (Serious) Adverse Events ... 45
10.2 Recording of (Serious) Adverse Events ... 46
10.3 Assessment of (Serious) Adverse Events ... 47
10.4 Reporting of Serious Adverse Events ... 48
10.5 Follow up of (Serious) Adverse Events ... 49
10.6 Potential risks/adverse events and treatment ... 50
10.7 Assessment of safety laboratory parameters ... 51
10.8 Pregnancy test ... 52
10.9 Physical examination ... 52
10.10 Exchange of safety data between sponsor and Rottapharm ... 52
11 DATA QUALITY ASSURANCE ... 53
11.1 Routine Monitoring ... 53
11.2 Audits and Inspections ... 53
11.3 Specification of Source Documents ... 54
12 STATISTICS ... 55
12.1 Statistical and Analytical Plans ... 55
12.2 Null and Alternative Hypotheses ... 55
12.2.1 Interim Analysis ... 55
12.2.2 Deviation(s) from the original statistical plan ... 55
12.3 Handling of Missing Data ... 55
12.4 Determination of Sample Size ... 55
13 DATA HANDLING AND RECORD KEEPING ... 56
14 CONFIDENTIALITY ... 57
15 INSURANCE ... 57
16 STUDY REGISTRATION ... 57
17 PUBLICATION POLICY ... 58
18 SIGNATURES ... 59
REFERENCES ... 62

1 LIST OF ABBREVIATIONS AND DEFINITIONS OF TERMS

AE	Adverse Event
ART	Antiretroviral Therapy
CRF	Case Report Form
eCRF	Electronic Case Report Form
cp	Copies
ETR	End of treatment response
EVR	Early virological response
GCP	Good Clinical Practice
HCC	Hepatocellular Carcinoma
HCV	Hepatitis C virus
HIV	Human immunodeficiency virus
IB	Investigator's Brochure
ICH	International Conference on Harmonization
IMP	Investigational Medicine Product
ISF	Investigator Site File
iSIL	Intravenous silibinin
ml	Milliliter
NR	Null responders
paR	Partial responders
PBMC	Peripheral blood mononuclear cells
PI	Principal Investigator
PI's	Protease inhibitors
PK	Pharmacokinetic
PR	Pegylated interferon and ribavirin
RPH	Rottapharm| Madaus
RVR	Rapid virological response
SAE	Serious Adverse Event
SDV	Source Data Verification
SHCS	Swiss HIV Cohort Study
SOC	Standard of care
SOP	Standard Operating Procedure
SUSAR	Suspected Unexpected Serious Adverse Reaction
SVR	Sustained virological response
TMF	Trial Master File
TT	Triple therapy

2 INVESTIGATORS AND STUDY ADMINISTRATIVE STRUCTURE

Sponsor

Jan Sven Fehr, MD
Attending physician
Division of Infectious Diseases and Hospital Epidemiology
University Hospital Zurich
University of Zurich
Rämistrasse 100
8091 Zurich
Email: jan.fehr@usz.ch
Phone: +41 44 255 34 02
Fax: +41 44 255 44 99

Study Sites:

University Hospital Zurich
Division of Infectious Diseases and Hospital Epidemiology
Rämistrasse 100
8091 Zurich

University Hospital Basel
Division for Infectious Diseases and Hospital Epidemiology
Petersgraben 4
4031 Basel

University Hospital Berne
Institute for Infectious Diseases
Friedbühlstrasse 51
3010 Berne

University Hospital Geneva
Division of Infectious Diseases
Rue Gabrielle-Perret-Gentil 4
1211 Geneva 14

University Hospital Lausanne
Division of Infectious Diseases
Rue du Bugnon 21
1011 Lausanne

Cantonal Hospital Sankt Gallen
Division of Infectious Diseases and Hospital Hygiene
Rorschacherstrasse 95
9007 Sankt Gallen

Regional Hospital Lugano
Via Tesserete 46

6900 Lugano

Principal investigators:

Zurich:
Dominique Laurent Braun, MD
Division of Infectious Diseases and Hospital Epidemiology
University Hospital Zurich
University of Zurich
Rämistrasse 100
8091 Zurich
Email: dominique.braun@usz.ch
Phone: +41 44 255 91 96
Fax: +41 44 255 44 99

Berne:
Andri Rauch, MD
University Hospital Berne
Institute for Infectious Diseases
Friedbühlstrasse 51
3010 Berne
Email: andri.rauch@insel.ch
Phone: +41 31 632 15 74

Basel:
Marcel Stöckle, MD
University Hospital Basel
Division for Infectious Diseases and Hospital Epidemiology
Petersgraben 4
4031 Basel
Email: stoecklem@uhbs.ch
Phone: +41 61 265 66 35

St. Gallen:
Dr. med. Patrick Schmid
Cantonal Hospital Sankt Gallen
Division of Infectious Diseases and Hospital Hygiene
Rorschacherstrasse 95
9007 St. Gallen
Email: patrick.schmid@kssg.ch
Phone: +41 71 494 10 28

Geneva:
Juan Ambrosioni, MD
University Hospital Geneva
Division of Infectious Diseases
Rue Gabrielle-Perret-Gentil 4
1211 Geneva 14

Email: juan.ambrosioni@hcuge.ch
Phone: +41 22 372 98 12

Lausanne:
Matthias Cavassini, MD
University Hospital Lausanne
Division of Infectious Diseases
Rue du Bugnon 21
1211 Geneva 14
Email: matthias.cavassini@chuv.ch.
Phone: +41 21 314 10 04

Lugano:
Enos Bernasconi, MD
Regional Hospital Lugano
Via Tesserete 46
6900 Lugano
Email: enos.bernasconi@eoc.ch
Phone: +41 91 811 64 90

Biometrician: Bruno Ledergerber, PhD
University Hospital Zurich
Division of Infectious Diseases and Hospital Epidemiology
Rämistrasse 100
8091 Zurich
Email: bruno.ledergerber@usz.ch
Phone: +41 44 255 33 57

Laboratory: Laurent Decosterd, PhD
Division of Clinical Pharmacology,
University Hospital Center
University of Lausanne
1011 Lausanne
Email: LaurentArthur.Decosterd@chuv.ch
Phone: +41 21 314 42 72

Data monitoring: Andreas U. Freiburghaus, MD
Head data management
Clinical trials center
University Hospital Zurich
Rämistrasse 100
8091 Zurich
Email: andreas.freiburghaus@usz.ch
Phone: +41 44 634 56 61

Clinical monitoring:	Clinical trials center<br>University Hospital Zurich<br>Rämistrasse 100<br>8091 Zurich<br>Email: andreas.freiburghaus@usz.ch<br>Phone: +41 44 634 56 61
Rottapharm:	Federica Girolami, PharmD, MSc<br>Corporate Pharmacovigilance and Drug Safety Department Director<br>Rottapharm Spa<br>Via Valosa di Sopra, 9<br>20900 Monza (MI), Italy<br>Email: federica.girolami@rottapharm.com<br>CDS@rottapharm.com (always to be kept in copy)<br>Phone: +39.039.7390395
	Lucio Rovati, M.D.<br>Chief Scientific Officer/Executive Medical Director<br>Rottapharm Spa<br>Via Valosa di Sopra, 9<br>20900 Monza (MI), Italy<br>Email: lucio.rovati@rottapharm.com<br>Phone: +39.039.7390318<br>Fax: +39.039.7390448

3 Ethics

3.1 Independent Ethics Committee (IEC) and Regulatory Authorities

Before this study will be conducted, the protocol, the proposed subject information and consent form as well as other study-specific documents will be submitted to a properly constituted Independent Ethics Committee (IEC) and regulatory authorities (Swissmedic/BAG) in agreement with local legal requirements, for formal approval. Any amendment to the protocol must be approved by these institutions.

The decision of the IEC and Swissmedic concerning the conduct of the study will be made in writing to the Sponsor-Investigator before commencement of this study.

3.2 Ethical Conduct of the Study

The study will be carried out in accordance with principles enunciated in the current version of the Declaration of Helsinki, the guidelines of Good Clinical Practice (GCP) issued by ICH, and Swiss regulatory authority's requirements.

IEC and regulatory authority will receive annual safety and interim reports and be informed about study stop/ end in agreement with local requirements.

3.3 Subject Information and Informed Consent

The investigator must explain to each subject the nature of the study, its purpose, the procedures involved, the expected duration, the potential risks and benefits and any discomfort it may entail. Each subject must be informed that the participation in the study is voluntary and that he/she may withdraw from the study at any time and that withdrawal of consent will not affect his/her subsequent medical treatment.

The subject must be informed that his/her medical records may be examined by authorized individuals other than their treating physician.

All subjects for this study will be provided a subject information sheet and a consent form describing this study and providing sufficient information for subjects to make an informed decision about their participation in this study.

The subject information sheet and the consent form will be submitted with the protocol for review and approval for the study by the IEC and by Swissmedic. The formal consent of a subject, using the approved consent form, must be obtained before that subject is submitted to any study procedure.

The subject should read and consider the statement before signing and dating the informed consent form, and should be given a copy of the signed document. The consent form must also be signed and dated by the investigator (or his designee) and it will be retained as part of the study records.

4 INTRODUCTION

This document is a protocol for a human research study.

4.1 Background and Rationale

Chronic hepatitis C virus (HCV) is a major cause of morbidity and mortality worldwide with an estimated number of 180 million infected patients. HCV infection causes around 376’000 deaths per year due to complications like hepatocellular carcinoma (HCC) and endstage liver disease (1). In Switzerland, 50’000-70’000 individuals are HCV infected (2). HCV-HIV co-infection is reported in approximately 30% of all Swiss HIV Cohort Study (SHCS) participants (3). In 2010, almost 100 liver transplantations have been performed, 25% of them because of HCV-related endstage liver disease (4).

Until 2012 the current standard treatment of patients with chronic hepatitis C was a 24 to 72 weeks therapy with pegylated inteferon-α and ribavirin (PR). Response to the treatment with PR depends on the HCV-genotype, HCV-HIV co-infection, baseline hepatitis C viral load, interleukin 28 B genotype, sex and stage of fibrosis (5). Sustained virological response (SVR) after treatment with PR can be achieved in 30% to 80% in treatment-naïve patients, depending on host and viral characteristics.

In 2012, the protease-inhibitors (PI’s) telaprevir and boceprevir as first directly acting HCV drugs have been approved by Swissmedic for hepatitis C mono-infected and HCV-HIV-co-infected individuals. However, therapy success is strongly limited in null-responders (NR) to previous PR. NR is defined as a reduction of < $2\log_{10}$ in HCV RNA after 12 weeks of therapy. Of note, in patients with previous failure to PR, TT is in fact a mono-therapy with a high risk of rapid development of resistance against the PI’s. Treatment of HCV-HIV co-infected individuals with the new PI’s is accompanied by additional challenges including drug-drug interactions, toxicity, and a high pill burden which impedes drug adherence.

Only preliminary data exist regarding the efficacy of a triple therapy (TT) with telaprevir or boceprevir and PR in co-infected patients. In a phase 2a study 59 HCV-HIV co-infected individuals with HCV genotype 1A were randomized to receive a TT for 12 weeks with telaprevir and PR followed by 36 weeks of PR compared with placebo and PR for 48 weeks. 70.5% of all patients treated with the TT achieved SVR compared to 34.4% in the placebo group (6). Importantly, in this study only treatment-naïve patients were included and less than 10% of patients had advanced fibrosis (≥ F3). However, patients with advanced fibrosis are at highest risk for decompensated liver disease and hepatocellular carcinoma (HCC) and prompt initiation of treatment is strongly recommended (5). Recently, data in mono-infected patients showed, that in prior non responders a 12 week course of a triple therapy (TT) with telaprevir and PR followed by another 24 weeks of PR resulted in an SVR of only 29% (6, 7). In HCV-HIV co-infected non-responders with unfavourable preconditions (e.g. HCV-genotype 1, interleukin 28 B non-CC genotype, advanced liver fibrosis, high baseline HCV viral load) SVR after TT is even expected to be lower. These patients urgently need additional therapeutic options with the goal to eradicate HCV in order to prevent further fibrosis progression and to reduce morbidity and mortality.

A promising substance in the field of drugs targeting the HCV replication is silibinin. Silibinin is the main component of silymarin, an extract of the milk thistle Silybum marinum. In 2008 Ferenci et al. for the first time reported the substantial clinical antiviral-effect of intravenous silibinin (iSIL) against HCV in PR non-responders. The administration of 20mg/kg iSIL in 20 patients led to a highly significant decrease in viral load (log drop 3.02±1.01) (18).

Figure 1: Changes in HCV RNA during IV administration of silibinin at various doses for 14 days, followed by combination therapy with PR, which was started on day 8. Ferenci et al., Gastroenterology, 2008

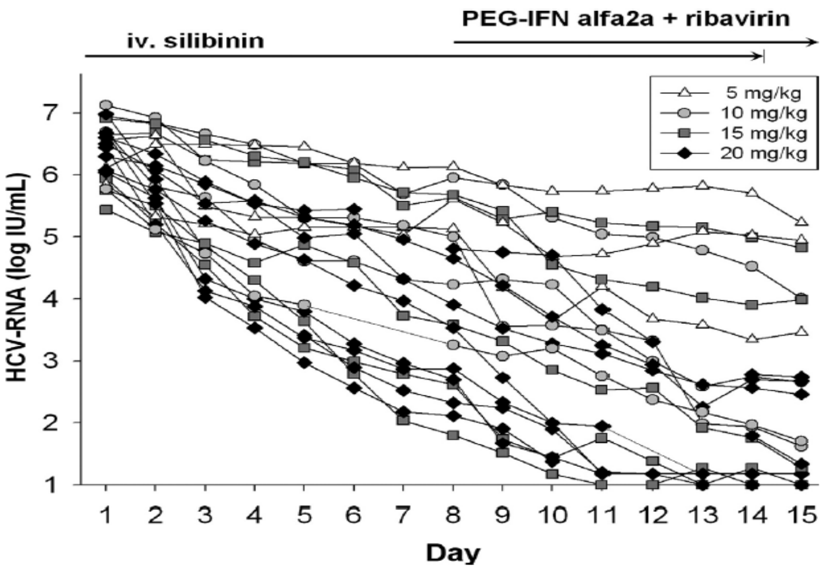

We intend to investigate the effect and tolerability of iSIL in HIV-HCV co-infected individuals with advanced liver fibrosis and previous non- or partial response to SOC.

4.2 Investigational Product

Intravenous Silibinin (iSIL) is available on the marked in its esterified modification silibinin-C-2, 3-dihydrogen succinate, disodium salt as Legalon® SIL. Legalon® SIL is currently authorized and marketed in Switzerland in the name of Max Zeller Söhne AG, licencee of Madaus GmbH a company of the Rottapharm| Madaus Group (RPH). Legalon® SIL is authorized since the mid-1980 for the treatment of hepatic intoxication with Amanita phalloides mushrooms in some EU Member states (e.g. in Germany since 18.04.1984). In Austria, Legalon® SIL is approved since December 2010 for the treatment of chronically infected HCV-patients with non- or partial response to standard therapy with PR. iSIL will be administrated intravenously during 2-4 hours.

Intravenous silibinin (iSIL) targets multiple steps in the virus life cycle and exhibits anti-oxidant, anti-inflammatory, anti-viral and immunomodulatory properties (8). iSIL inhibits the HCV NS5B polymerase activity directly or by interfering with the binding of RNA to this enzyme (9). In addition, iSIL appears to block virus entry, virus transmission and virus secretion (10).

4.3 Preclinical Data

Detailed information about Non-Clinical studies are provided in the Investigator Brochure, which are briefly summarized hereafter

Safety Data:
In the acute test silibinin-C-2´, 3-dihydrogen succinate (SHS), disodium salt proved to be practically non-toxic. The LD50 values after intravenous injection may thus be accepted for rats and mice of both sexes as > 1000 mg/kg.
After intravenous, intra-arterial or intramuscular injection a good local tolerance was observed in rats and rabbits.
In a subacute study over 4 weeks, the active ingredient was well tolerated by male and female beagle hounds in the dose range investigated up to 150 mg/kg per infusion – except for a low-grade transient depressant effect on the circulation in the high dosage group.
Toxicological investigations into the reproduction of rats and rabbits did not reveal any embryo-lethal and/or teratogenic effects after doses of up to 50 mg/kg. After the administration of higher doses, fetal death due to maternal toxic effects was recorded.
Mutagenicity tests carried out on microorganisms (Ames test) and mammalian cells in vitro (CHO- and Mouse-Lymphoma Test) did not raise concerns.
In a more recent preclinical toxicity study, a significant and reversible increase in total bilirubin was observed in dogs at toxicological doses (200/400 mg/kg expressed as SHS). No other changes related to liver dysfunction and no abnormal histopathological findings were observed. No signs of cholestasis were detected histologically.

Pharmacodynamic properties:
The antiviral effects of SHS were evaluated in a biochemical assay for inhibitors of NS5B RNA-dependent RNA polymerase, in HCV genotype 1a and 1b replicon systems, and in a HCV genotype 2a strain JFH1 cell culture system. These studies showed that SHS is able to inhibit HCV N5SB polymerase function and HCV replication at concentrations that are in the same order of magnitude of the SHS concentration that can be measured in the plasma of HCV patients after intravenous administration of Legalon® SIL at doses equivalent to 20mg silibinin per kg body weight.

4.4 Clinical Data to Date

The clinical effect of iSIL on HIV replication was described in one case of a HIV-HCV co-infected patient (11). The CD4 count of this patient was 390/ul and the patient had no ART. At the start of intravenous treatment with 20mg/kg iSIL, HCV viral load was 126’000 IU/ml and HIV viral load 1’830 IU/ml. After one week of iSIL mono-therapy, HCV-RNA decreased to 78 IU/ml and HIV-RNA decreased to 640copies/ml. Then combination-therapy with PR was added and iSIL was continued in the same dosage. After 14 days, both HCV-RNA and HIV-RNA were undetectable. At 24 weeks after the end of therapy HCV-RNA was still negative. Tolerability of iSIL was excellent without any adverse events. The efficacy of iSIL as rescue-therapy in on-treatment non-responders to full dose of PR was reported by Rutter at al. (12). In this study the administration of iSIL in 27 patients with previous non response to PR led to undetectable HCV-RNA in 85% after 21 days and 7/11 (63.6%) reached SVR. In this patient group a high proportion had advanced liver fibrosis (70% METAVIR fibrosis

score ≥ 3), an unfavourable interleukin 28 B genotype and a high baseline HCV viral load. The study results confirmed the efficacy of iSIL in a difficult-to treat patient group and underscored that iSIL could be a valuable treatment-option in patients not responding to PR (12). The potential role of iSIL as rescue therapy in patients not responding to previous combined therapy with PR and a HCV-specific PI was demonstrated by Biermer et al., 9 of the 13 responder patients are in the follow-up period: 3 of them achieved SVR24, 2 achieved SVR12, and 4 had a viral relapse (13). Recent reports also show the efficacy of iSIL in patients with HCV-reinfection after liver transplantation. After 14 days treatment of iSIL in a dosage of 20mg/kg all four patients showed normalization of liver enzymes and a significant decline of HCV-RNA with a mean log-reduction of 2.8 (14). Moreno et al. presented their data about the safety and the anti-HCV activity of a rescue therapy with iSIL in cirrhotic HIV-HCV co-infected individuals at the 19th conference on retroviruses and opportunistic infections (CROI) in 2012. They treated 4 individuals with previous partial response (PR) or relapses to PR with iSIL 20mg/kg for at least 14 days. Three patients started iSIL during PR and nitazoxanide (add-on strategy). Overall, therapy with iSIL led to negative HCV-RNA in 3 subjects and values <50 IU/mL in the remaining. The period of mono-therapy with iSIL produced a time-dependent effect: the mean decrease of HCV-RNA was –2.5 log_{10} IU/ml after 1 week and –4.62 log_{10} IU/ml after 35 days. However, HCV-RNA rebounded in 4 patients after stopping iSIL. There were no significant adverse events related to iSIL and all patients maintained current antiretroviral therapy (ART) with undetectable HIV RNA (15).

4.5 Dose Rationale

All included study-subjects will receive a lead-in therapy with iSIL in a dosage of 20mg/kg/day (expressed as silibinin concentration) once a day for 14 days. At the end of the THISTLE study, i.e. after the day of completion of the 14-day iSIL administration (day 15), the patients will be considered for eligibility to receive standard of care TT with telaprevir (i.e. 12 weeks combination therapy with telaprevir plus PR followed by 36 weeks of PR).
The rationale of 20mg/Kg/day is the observation, that there exist a strong dose-dependent effect with a greatest HCV-RNA decline when iSIL is administrated in a dose of 20mg/kg, whereas the lower dose (5, 10 or 15mg/kg) led to a significant lower HCV-RNA decline (16). iSIL dose-dependently reduced and even completely eradicated the HCV load, with the dose of 20 mg/kg per day being clearly the most effective dose.

Since effective antiviral concentration cannot be reached with the oral administration with the available formulations of silymarin/silibinin, the drug needs to be administrated intravenously. The rationale to use 14 days of treatment is a trade-off between virological efficacy of this treatment and logistic and financial constraints. Previous reports (10, 16) demonstrated a continuous decline during 2 weeks of iSIL therapy with a mean decline of 3 logs IU/ml in HCV-RNA. We assume that this decline would substantially improve the chances of SVR as this reduction of viral load should both increase the efficacy of PR and reduce the odds of drug resistance to telaprevir.

4.6 Risk/Benefits and Ethical Considerations

The study has not been designed as placebo-controlled study because of ethical considerations. A placebo-controlled study was extensively discussed in our scientific-

group, within SHCS and also representatives of the ethical committee of the University of Zurich. On the background of the urgent need of a new treatment strategy for this selected difficult to treat patient population together with the apparently excellent tolerability of iSIL, it was considered ethically not justifiable to withhold the lead-in treatment with iSIL, and as a consequence it was decided to offer all patients the chance to receive an active treatment since a standard rescue strategy is not available for the treatment of patients with incomplete virological response.

In addition, the expected benefits of iSIL justify the risks, which possibly arise from the administration of the study medication. Details about the risks associated with iSIL administration have been provided in Section 10.6. Basically this study is considered to be a phase II trial with focus to safety and tolerability.

5 STUDY OBJECTIVES AND OUTCOMES

5.1 Primary Variable(s)

Primary aims

The aim of this study is to evaluate the safety and efficacy of a 14 days lead-in course with iSIL in HCV-HIV co-infected individuals who failed previous treatment with PR.

- To assess the safety of iSIL in the context of HCV-HIV co-infected patients and HIV antiretroviral treatment (ART).
- To evaluate the kinetics of the decline in HCV RNA from baseline after 2 weeks of iSIL treatment

Primary endpoints:

- Frequency of adverse events during iSIL treatment.
- Kinetics of the decline in HCV-RNA after 2 weeks of iSIL treatment (difference in IU/ml from day 1 to day 15, see flow-chart below).

5.2 Secondary Variables

Secondary aims

- To assess the efficacy of iSIL in a variety of clinically meaningful virological responses
- To evaluate drug levels of iSIL and to assess its influence on the drug-levels of co-administrated ART.

Secondary endpoints:

- Drug levels of iSIL and its influence on the drug-level of co-administrated ART.
- Proportion of patients with HIV virological failure, i.e. confirmed viremia >50cp/ml.

6 STUDY DESIGN AND COURSE OF STUDY

6.1 General Design

This study is a multicentre, open-label, interventional study including HCV-HIV co-infected individuals with chronic HCV-genotype 1 infection and documented previous null-response (NR) or partial-response (paR) to standard therapy with SOC and progressed liver fibrosis.

The overall study-duration is 15 days. The first part is the once a day administration of iSIL from day 1 to 14. Since the half-life (T ½) of the administrated study-drug is very short (3 hours), the follow-up of the study-subjects is one day (day 15) and the final visit will be on day 15.
Subsequent to THISTLE (at day 15) we have the intention to include the study-subjects in the SHCS trial # 688. The trial # 688 will investigate the occurrence of HCV-resistance to the HCV-PIs during TT. The duration of the # 688 will contain 48 weeks with a follow-up of 24 weeks. Importantly, we plan to review the results from the THISTLE trial with the data from the SHCS # 688.

The estimated time frame is listed below:

- Submission to ethical committee (Zurich as lead ethical committee): November 2012
- Ethical approval: January 2013
- Swissmedic approval: February 2013
- First patient enrolled: April 2013
- Enrolment: April 2013 to December 2013
- Last patient enrolled: End of December 2013

All included patients will receive a lead-in therapy with iSIL in a dosage of 20mg/kg/day for 14 days followed by standard of care TT. As mentioned above, the follow-up period will contain for one day due to short half-life time of the administrated study-drug. Moreover, a prolonged follow-up period outside THISTLE trial is warranted due to the SHCS trial # 688.

The included patients are HCV-HIV co-infected SHCS participants with chronic HCV-genotype 1 infection and documented previous null-response (NR) or partial-response (paR) to standard therapy with SOC and in urgent need of treatment (i.e. METAVIR fibrosis score ≥2 or ISHAK fibrosis score ≥4 or fibroscan stiffness ≥ 9.5kPa). As cut-offs for the fibroscan we used the ones proposed in a recent review by Castera el al (17). NR is defined as less than 2 log10 IU/ml decrease in HCV RNA level from baseline at 12 weeks. paR is defined as more than 2 log10 IU/ml decrease in HCV-RNA level from baseline at 12 weeks of therapy but detectable HCV-RNA at weeks 12 and 24.

A minimum of 20 study-subjects will be included based on our calculation (see below).

Importantly, we will not randomize patients to either lead-in phase with iSIL or placebo before starting TT because of ethical considerations. On the background of the excellent tolerability of iSIL together with the urgent need of a new treatment strategy for this selected difficult to treat patient population, it is not justifiable to withhold the lead-in treatment with iSIL.

6.2 Study visits

The study procedures are in detail mentioned below. We refer to the chapters 9.1. - 9.19

7 SUBJECT SELECTION AND WITHDRAWAL

7.1 Inclusion Criteria

HCV-HIV co-infected SHCS participants with chronic HCV-genotype 1 infection and documented previous null-response (NR) or partial-response (paR) to standard therapy with SOC and in urgent need of treatment (i.e. METAVIR fibrosis score ≥ 2 or ISHAK fibrosis score ≥4 or fibroscan stiffness ≥ 9.5kPa) will be included. As cut-offs for the fibroscan we used the ones proposed in a recent review by Castera el al (19). NR is defined as less than 2 log10 IU/ml decrease in HCV RNA level from baseline at 12 weeks. paR is defined as more than 2 log10 IU/ml decrease in HCV-RNA level from baseline at 12 weeks of therapy but detectable HCV-RNA at weeks 12 and 24.

Subjects will undergo an informed consent process in accordance with GCP. To qualify for the study, subjects must meet all inclusion and none of the exclusion criteria

Inclusion criteria:

- Male and Female patients ≥ 18 years
- HIV-HCV co-infection
- HCV Genotype 1 infection
- At least one liver biopsy since diagnosis of HCV-infection
- Fibrosis score METAVIR ≥ 2 documented by biopsy OR a stiffness ≥ 7.0kPa documented by fibroscan during the previous 12 months.
- Documented previous null-response or partial-response to SOC
- Signed Informed Consent after being informed

7.2 Exclusion Criteria

The presence of any one of the following exclusion criteria will lead to exclusion of the subject:

Exclusion criteria:

- contraindications to the study drug under study, e.g. known hypersensitivity or allergy to any ingredient of the study drug,
- women who are pregnant or breast feeding,
- intention to become pregnant during the course of the study,
- lack of safe contraception, defined as:

  Female subjects of childbearing potential, not using and not willing to continue using a medically reliable method of contraception for the entire study duration, such as oral, injectable, or implantable contraceptives, or intrauterine contraceptive devices,

or who are not using any other method considered sufficiently reliable by the investigator in individual cases.

Please note that female subjects who are surgically sterilized/hysterectomized or post-menopausal for longer than 2 years are not considered as being of child bearing potential.

- Male patients who do not agree to abstain from intercourse or who do not use a condom
- other clinically significant concomitant unstable disease states (e.g., renal failure, unstable cardiovascular disease),
- known or suspected non-compliance, drug or alcohol abuse,
- inability to follow the procedures of the study, e.g. due to language problems, psychological disorders, dementia, etc. of the subject,
- participation in another study with investigational drug within the 30 days preceding and during the present study,
- previous enrolment into the current study,
- enrolment of the investigator, his/her family members, employees and other dependent persons
- Patients in need of ART with HIV virological failure (≥ 400 copies/ml) in the last 3 months
- Any other condition that, in the opinion of the Investigator, may jeopardize the study conduct according to the protocol.

7.3 Subject Recruitment and Screening

Eligible patients were selected by a review of the SHCS database (Download April 2012) using following criteria: i) HCV infection, ii) HCV genotype 1, iii) previous treatment with PR, iv) detectable HCV viral load after treatment. 80 potential eligible patients have been identified. The principal investigators of each study site (Basel, Berne, Geneva, Lausanne, Lugano, St. Gallen, and Zurich) will check the charts of their patients regarding meeting the inclusion/exclusion criteria. In the SHCS center Zurich 24 of the selected 80 patients are treated. We checked the electronic charts of these 24 patients and found 7 patients (~25%) meeting the definitive main inclusion criteria (e.g. METAVIR fibrosis score ≥ 2, previous NR or paR). Accordingly we used the 25% rate for every center to estimate the minimal number of individuals who can potentially be included in this study (Tab.1).

Table 1: Minimal estimated number of individuals potentially eligible for therapy with Silibinin in the SHCS at present

**Centers**	**Number of patients**
Basel	3
Berne	4
Geneva	3
Lausanne	2
Lugano	1
St. Gallen	2
Zurich	5
**TOTAL**	**20**

The study participants will receive no payment as compensation for time and effort. Since the triple therapy is standard of care, the costs of PR and telaprevir are covered by the patients insurance.

7.4 Early Withdrawal of Subjects

The subject has to be withdrawn from the study prior the expected completion if there is a failure of the subject to adhere to protocol requirements.

Any subjects experiencing a serious adverse event felt to be related to study drug should be withdrawn from the study. Subjects will be withdrawn if they require hospitalization for addiction treatment. Any subject withdrawing their consent to participate in the study will be withdrawn from the study. Subjects discontinued from the clinical trial will be scheduled for a final evaluation and given appropriate treatment referrals. Subjects withdrawn may be replaced, at the discretion of the principal investigator.

Subjects will be informed at the consent session that treatment may be discontinued due to:

- The subject withdraws his/her informed consent to participate in the study
- Intolerable side effects
- Significantly abnormal laboratory values confirmed with a repeat testing
- Clinical deterioration for any reason or any clinical status that necessitates hospital admission
- Lost to follow up
- Protocol Violation

Reasons why subjects are discontinued from the clinical trial will be documented on the Study Termination Form, along with any referrals that are made. A final safety evaluation will be conducted as soon as possible on all treated subjects who are discontinued. This will include study-required laboratory testing, physical examination, and urine pregnancy test (women only).

7.5 Early Termination of Study

The Sponsor may terminate the study prematurely according to certain circumstances:

- insufficient patient recruitment,
- when the safety or benefit of the subjects is doubtful or at risk, respectively,
- alterations in accepted clinical practice that make the continuation of a clinical trial unwise

8 TREATMENTS

8.1 Treatments to be administered

iSIL is administrated in its esterified modification silibinin-C-2, 3-dihydrogen succinate disodium salt as Legalon® SIL. iSIL is administrated intravenously at the first day during 4 hours and, if tolerated, from the second day during 2 hours and, if tolerated, from the third day during 1 hour. The dosage is 20mg/kg once daily for 14 days. The preparation of iSIL is done by the study-nurse.

<u>Preparation of the Legalon® SIL solution:</u>

1) The patient's body weight (in kilogram) is multiplied by a factor 20 (corresponding to the administrated dose of 20mg/kg) obtaining the milligrams to be infused (e.g. 100 kg x 20 mg/kg = 2000 mg).
2) The calculated number of milligrams are divided trough 350 (corresponding to the number of milligrams of silibinin contained in 1 vial Legalon® SIL). The result indicates the number of vials Legalon required to be diluted.
3) For every Legalon® SIL vial a syringe with 35ml sodium chloride 0.9% (NaCl 0.9%) is drawn up from a single infusion bottle of NaCl 0.9% and are injected in every single vial Legalon. Every vial contains now a solution of 350mg Legalon® SIL with a solution concentration of 10 mg/ml.
4) The total ml of the Legalon® SIL solution (10 mg/ml) will be calculated, dividing the milligrams obtained at point 1 per 10 (e.g. 2000mg / 10mg/ml = 200ml).
5) The total millilitres of the Legalon® SIL-NaCl 0.9% solution will be infused to the patients with a constant flow.
6) The final solution will be put into an infusion bag that is to be identified with a tear-off label supplied by RPH.

No more than 6 hours (below 30°C) should elapse once the Legalon®SIL-NaCl 0.9% solution is prepared until the end of the administration to the patient. The Legalon® SIL-NaCl 0.9% solution will be administered as scheduled to last about 1-4 hours with a constant flow.

8.2 Identity of Investigational Product(s)

Intravenous Silibinin (iSIL) is available on the marked in its esterified modification silibinin-C-2, 3-dihydrogen succinate and disodium salt as Legalon® SIL. Legalon is currently authorized and marketed in Switzerland in the name of Max Zeller Söhne AG, licencee of Madaus GmbH a company of the Rottapharm| Madaus Group (RPH). Legalon® SIL is authorized since the mid-1980 for the treatment of hepatic intoxication with *Amanita phalloides* mushrooms in some EU Member states (e.g. in Germany since 18.04.1984). In Austria, Legalon® SIL is approved since December 2010 for the indication "Concurrent medication for patients with chronic hepatitis C who do not respond (sufficiently) to standard therapy with pegIFN / ribavirin (non-responders)". In Switzerland, Legalon® SIL has not yet been approved for treatment of chronic HCV.

8.3 Method of Assigning Subjects to Treatment Groups

There is only one treatment group with Legalon® SIL, so no randomization or stratification methods will be implemented in this trial.

See section 4.6 for details.

8.4 Treatment Compliance

Since the study medication (iSIL) is administrated by intravenous route surveyed by the study nurse every day in the outpatient unit, the adherence should be warranted.

The patient compliance will be evaluated counting the related labels attached on the drug inventory/dispensing record and the unused boxes remaining at the trial site.

The site will be provided with a set of tear off labels; when the study medication is prepared in the site/Pharmacy, the label will be stuck on the drip/bag; before treatment administration the tear-off part of the label applied on the bag will be removed by the investigator and it will be stuck on the drug inventory/dispensing record.

8.5 Prior and Concomitant Therapy

All concomitant and/or rescue treatment(s) have to be recorded in the eCRF.

Potential and harmful interactions of concomitant drugs as required dose adjustments of co-medication have to be checked by the principal investigator during the screening visit using following drug-interaction databank: www.hiv-druginteraction.com.
If clinically indicated, drug levels of concomitant drugs have to be measured before first administration of iSIL.

8.6 Packaging, Labelling and Supply

Legalon® SIL packages will be provided to the site as pre-packaged commercially available products (re-labelled as appropriate for the clinical trial). Each carton box of Legalon® SIL contains 4 vials. One Legalon® SIL vial contains 350 mg of silibinin (315 mg according HPLC determination) corresponding to 528.5 mg silibinin-C-2 ',3-dihydrogensuccinate disodium salt (corresponding to 476 mg of mono dihydrogensuccinate sodium salts determined by HPLC method).

All packaging and labeling operations will be performed by RPH according to GMP and GCP requirements.
The test drug will be supplied as a sterile, endotoxin-free lyophilized powder for intravenous infusion in amber glass vials, packed in boxes containing four vials each.

Both vials and boxes will be labeled in English and in local language with at least the following information:
- Protocol/ Study No.
- Batch No.
- Pharmaceutical form, Dosage, Route of administration
- Sponsor Name
- Investigator Name, address and telephone number
- Storage instructions

- Expiry date
- Directions for use
- "For clinical trial use only"

Sample labels will be filed in the Trial Master File (TMF) by the Sponsor.
RPH will provide the Study drugs to each site together with the relevant documentation.
The Legalon® SIL shipments will be performed by an authorised courier that will ensure continuous monitoring of the shipment temperature. The temperature under 25°C should be maintained for transport. Records of temperature measures, throughout the whole shipment period, will be provided by the courier and filed in the appropriate Trial Master File section and a copy in the Investigator’s File.
After reception the Site staff must complete and return to RPH the IMP Supply Shipping/Receipt Form, verifying the receipt and integrity of the test drug. RPH will retain reference samples of the test drug.
Responsibility for Investigational Product(s) accountability at the trial site rests with the Principal Investigator or other adequately authorized delegate under the supervision of the Principal Investigator. The Site study staff should maintain records of IMP delivery, inventory at the site, use by each patient, and return or alternative disposition for unused products.
For all study medications, a numbering system in accordance with all requirements of GMP will be used. This will ensure that any dose of study medication can be identified and traced back to the original bulk ware/batch of the active ingredients.

8.7 Blinding

Since there is no comparator-arm, this trial will be conducted open-label where the treatment assignments are known to the investigator and the patient.

8.8 Storage Conditions

Drug supplies must be kept in a secure, limited access storage area, in their original packaging and under the recommended storage conditions defined below. Legalon® SIL should be stored under 25°C. Storage temperature should be monitored by electronic min/max calibration certificated thermometers and documented once weekly on temperature log
The Principal Investigator should ensure the safe and secure IMP storage according to specifications provided by RPH and the use strictly in accordance with the study protocol.

8.9 Study Drug Accountability

The principal investigator of each study site must maintain accurate and adequate records including dates, lot number, quantities received and individual usage.

IMP inventory and dispensing will be documented as follows:
-An IMP Inventory Form will be maintained and updated by the Site study staff; it will document the amounts received, dispensed, returned or unused.

-An IMP Accountability Form will be maintained and updated for all patients included by Investigator; it will document the amounts dispensed to each subject.

8.10 Return or Destruction of Study Drug

At the completion of the study, there will be a final reconciliation of drug shipped, drug consumed, and drug remaining. This reconciliation will be logged on the drug accountability form, signed and dated. Any discrepancies noted will be investigated, resolved, and documented prior to return or destruction of unused study drug. The Site study staff should return the partially used and unused study drug to RPH.

9 STUDY PROCEDURES

9.1 Day – 14 (Screening visit)

- Information of patient and Informed Consent
- Check for inclusion and exclusion criteria
- Medical History and prior concomitant medication
- Physical Examination:
  - Including blood pressure while sitting (right/left), pulse rate, temperature (degree Celsius), cardiac auscultation, chest auscultation, abdominal auscultation, brief neurological examination
- Concomitant medication
- Pregnancy test:
  - 10ml Heparin blood
- HCV-RNA, HIV-RNA
  - 10ml EDTA blood
- Full blood count, electrolytes (sodium, potassium), ASAT, ALAT, total and fractionated bilirubin, alcaline phosphatase, gammaGT, creatinine, albumin, total protein, INR, thyroid stimulating hormone (TSH)
  - 10ml Heparin blood
- Interleukin 28B genotype (if not available):
  - 10ml EDTA blood
- Plasma aliquot as backup-blood for potential later analysis
  - 10ml ETDA blood
- Accepted time window: ± 3 days

9.2 Day + 1 (baseline visit), immediately previous to iSIL administration

- Check for inclusion and exclusion criteria
- Documentation of adverse events
- Concomitant medication
- Physical examination
  - Including blood pressure while sitting (right/left), pulse rate, temperature (degree Celsius), cardiac auscultation, chest auscultation, abdominal auscultation, brief neurological examination

- Safety laboratory: full blood count, electrolytes (sodium, potassium), ASAT, ALAT, total and fractionated bilirubin, gammaGT, total protein, alcaline phosphatase, creatinine, albumin, INR
    - 10ml Heparin blood
- HCV-RNA, HIV-RNA
    - 10ml EDTA blood
- CD3/4/8 count
    - 3ml EDTA blood
- Drug level of current ART
    - 10ml EDTA blood
    - Note the time-point (date, hour, minutes) of the last intake and the dosage (milligram) on the electronic CRF
    - Blood (10 ml) must be collected in EDTA solution vacutainer tubes. The tubes containing EDTA solution should be stored at +4°C. Tubes must be identified with the patient ID number and sample number as soon as blood has been collected
    - Blood samples must be brought to the laboratories without delay (maximum 30 min after blood collection).
    - Treatment of samples: centrifugation at 1500g for 10 min at +4°C. In a class II biohazard hood plasma (obtained after centrifugation) should be divided in three labelled tubes (of approximately 1mL each) ready for freezing at -80 ° C, within one hour after collection. If this deadline cannot be met, please keep the sample at 4° C until centrifugation and freezing
- Silibinin drug-level (Cmin (T0, pre-dose), Cmax (i.e immediately after the end of silibinin infusion) and one sampling at T4 (i.e. 4 hour after the end of silibinin infusion)
    - Blood (6 ml) must be collected in ACD (acid citrate dextrose) solution vacutainer tubes. The tubes containing ACD solution should be stored at +4°C. Tubes must be identified with the patient ID number and sample number as soon as blood has been collected
    - Blood samples must be brought to the laboratories without delay (maximum 30 min after blood collection).
    - Treatment of samples: centrifugation at 1500g for 10 min at +4°C. In a class II biohazard hood plasma (obtained after centrifugation) should be divided in three labelled tubes (of approximately 1mL each) ready for freezing at -80 ° C, within one hour after collection. If this deadline cannot be met, please keep the sample at 4° C until centrifugation and freezing.
- Plasma aliquot as backup-blood for potential later analysis
    - 10ml ETDA blood
- PBMC: only at the study site Zurich

    - 10ml Heparin blood
- Administration of iSIL 20mg/Kg during 4 hours
    - Preparation of solution see below in the protocol
    - Vital parameters before, every 15min and after administration of iSIL
    - Documentation of side effects in the electronic CRF
    - If a central venous catheter (cvc) has to be placed due to poor condition of the peripheral veins:
        - The indication for cvc must be done by the principal-investigator
        - The correct placement of the catheter must be documented by X-ray or using the ECG-triggering method and documented in the electronic medical history
        - The insert site of the cvc must be checked every visit by the study nurse and signs of an infection must be documented in the CRF
        - If there are signs of an infection the cvc must be removed immediately and the catheter tip must be send to the microbiological laboratory
    - If a peripheral venous catheter has placed:
        - The Investigators are recommended to strictly adhere to the "Guidelines for the Prevention of Intravascular Catheter-Related Infections" (20)

9.3 Day 2 (visit 3)

- Documentation of adverse events
- Concomitant medication
- Physical examination
    - Including blood pressure while sitting (right/left), pulse rate, temperature (degree Celsius), cardiac auscultation, chest auscultation, abdominal auscultation, brief neurological examination)
- Safety laboratory: full blood count, electrolytes (sodium, potassium), ASAT, ALAT, total and fractionated bilirubin, gamma GT, alcaline phosphatase, creatinine, albumin, total protein, INR
    - 10ml Heparin blood
- HCV-RNA,
    - 10ml EDTA blood
- Silibinin drug-level T0 (pre-dose): only at the study sites Zurich and Lausanne
    - Blood (6 ml) must be collected in ACD (acid citrate dextrose) solution vacutainer tubes. The tubes containing ACD solution should be stored at +4°C. Tubes must be identified with the patient ID number and sample number as soon as blood has been collected

- Blood samples must be brought to the laboratories without delay (maximum 30 min after blood collection).
- Treatment of samples: centrifugation at 1500g for 10 min at +4°C. In a class II biohazard hood plasma (obtained after centrifugation) should be divided in three labelled tubes (of approximately 1mL each) ready for freezing at -80 ° C, within one hour after collection. If this deadline cannot be met, please keep the sample at 4° C until centrifugation and freezing.

- Administration of iSIL 20mg/Kg during <u>2 hours</u>
  - Preparation of solution see below in the protocol
  - Vital parameters before, every 15min and after administration of iSIL
  - Documentation of side effects in the electronic CRF
- Accepted time window: same day

9.4 Day 3 (visit 4)

- Documentation of adverse events
- Concomitant medication
- HCV-RNA,
  - 10ml EDTA blood
- Administration of iSIL 20mg/Kg during <u>1 hour</u>
  - Preparation of solution see below in the protocol
  - Vital parameters before, every 15min and after administration of iSIL
  - Documentation of side effects in the electronic CRF
- Accepted time window: same day

9.5 Day 4 (visit 5)

- Documentation of adverse events
  - use the electronic CRF
- Concomitant medication
  - use the electronic CRF
- Physical examination
  - Including blood pressure while sitting (right/left), pulse rate, temperature (degree Celsius), cardiac auscultation, chest auscultation, abdominal auscultation, brief neurological examination

- Safety laboratory: full blood count, electrolytes (sodium, potassium), ASAT, ALAT, total and fractionated bilirubin, gamma GT, alcaline phosphatase, creatinine, albumin, total protein, INR
    - 10ml Heparin blood
- HCV-RNA,
    - 10ml EDTA blood
- Silibinin drug-level T0 (pre-dose): only at the study sites Zurich and Lausanne
    - Blood (6 ml) must be collected in ACD (acid citrate dextrose) solution vacutainer tubes. The tubes containing ACD solution should be stored at +4°C. Tubes must be identified with the patient ID number and sample number as soon as blood has been collected
    - Blood samples must be brought to the laboratories without delay (maximum 30 min after blood collection).
    - Treatment of samples: centrifugation at 1500g for 10 min at +4°C. . In a class II biohazard hood plasma (obtained after centrifugation) should be divided in three labelled tubes (of approximately 1mL each) ready for freezing at -80 ° C, within one hour after collection. If this deadline cannot be met, please keep the sample at 4° C until centrifugation and freezing.
- Administration of iSIL 20mg/Kg during 1 hour
    - Preparation of solution see below in the protocol
    - Vital parameters before, every 15min and after administration of iSIL
    - Documentation of side effects in the electronic CRF
- Accepted time window: same day
-

9.6 Day 5 (visit 6)

- Documentation of adverse events
- Concomitant medication
- HCV-RNA,
    - 10ml EDTA blood
- Administration of iSIL 20mg/Kg during 1 hour
    - Preparation of solution see below in the protocol
    - Vital parameters before, every 15min and after administration of iSIL
    - Documentation of side effects in the electronic CRF
- Accepted time window: same day

9.7 Day 6 (visit 7)

- Documentation of adverse events
- Concomitant medication
- HCV-RNA,
  - 10ml EDTA blood
- Administration of iSIL 20mg/Kg during 1 hour
  - Preparation of solution see below in the protocol
  - Vital parameters before, every 15min and after administration of iSIL
  - Documentation of side effects in the electronic CRF
- Accepted time window: same day

9.8 Day 7 (visit 8)

- Documentation of adverse events
- Concomitant medication
- HCV-RNA,
  - 10ml EDTA blood
- Administration of iSIL 20mg/Kg during 1 hour
  - Preparation of solution see below in the protocol
  - Vital parameters before, every 15min and after administration of iSIL
  - Documentation of side effects in the electronic CRF
- Accepted time window: same day

9.9 Day 8 (visit 9)

- Documentation of adverse events
  - use the electronic CRF
- Concomitant medication
  - use the electronic CRF
- Physical examination
  - Including blood pressure while sitting (right/left), pulse rate, temperature (degree Celsius), cardiac auscultation, chest auscultation, abdominal auscultation, brief neurological examination

- Safety laboratory: full blood count, electrolytes (sodium, potassium), ASAT, ALAT, total and fractionated bilirubin, gamma GT, alcaline phosphatase, creatinine, albumin, total protein, INR
    - 10ml Heparin blood
- HCV-RNA
    - 10ml EDTA blood
- Silibinin drug-level T0 (pre-dose): only at the study sites Zurich and Lausanne
    - Blood (6 ml) must be collected in ACD (acid citrate dextrose) solution vacutainer tubes. The tubes containing ACD solution should be stored at +4°C. Tubes must be identified with the patient ID number and sample number as soon as blood has been collected
    - Blood samples must be brought to the laboratories without delay (maximum 30 min after blood collection).
    - Treatment of samples: centrifugation at 1500g for 10 min at +4°C. In a class II biohazard hood plasma (obtained after centrifugation) should be divided in three labelled tubes (of approximately 1mL each) ready for freezing at -80 ° C, within one hour after collection. If this deadline cannot be met, please keep the sample at 4° C until centrifugation and freezing.
- Plasma aliquot as backup-blood for potential later analysis
    - 10ml ETDA blood
- PBMC: only at the study site Zurich
    - 10ml Heparin blood
- Administration of iSIL 20mg/Kg during <u>1 hour</u>
    - Preparation of solution see below in the protocol
    - Vital parameters before, every 15min and after administration of iSIL
    - Documentation of side effects in the electronic CRF
- Accepted time window: same day

9.10 Day 9 (visit 10)

- Documentation of adverse events
- Concomitant medication
- HCV-RNA
    - 10ml EDTA blood
- Administration of iSIL 20mg/Kg during <u>1 hour</u>
    - Preparation of solution see below in the protocol
    - Vital parameters before, every 15min and after administration of iSIL

    - Documentation of side effects in the electronic CRF
- Accepted time window: same day

9.11 Day 10 (visit 11)

- Documentation of adverse events
- Concomitant medication
- HCV-RNA,
    - 10ml EDTA blood
- Administration of iSIL 20mg/Kg during <u>1 hour</u>
    - Preparation of solution see below in the protocol
    - Vital parameters before, every 15min and after administration of iSIL
    - Documentation of side effects in the electronic CRF
- Accepted time window: same day

9.12 Day 11 (visit 12)

- Documentation of adverse events
- Concomitant medication
- HCV-RNA,
    - 10ml EDTA blood
- Administration of iSIL 20mg/Kg during <u>1 hour</u>
    - Preparation of solution see below in the protocol
    - Vital parameters before, every 15min and after administration of iSIL
    - Documentation of side effects in the electronic CRF
- Accepted time window: same day

9.13 Day 12 (visit 13)

- Check for inclusion and exclusion criteria
    - use electronic CRF
- Documentation of adverse events
    - use the electronic CRF
- Concomitant medication
    - use the electronic CRF

- Physical examination
    - Including blood pressure while sitting (right/left), pulse rate, temperature (degree Celsius), cardiac auscultation, chest auscultation, abdominal auscultation, brief neurological examination
- Safety laboratory: full blood count, electrolytes (sodium, potassium), ASAT, ALAT, total and fractionated bilirubin, gamma GT, alcaline phosphatase, creatinine, albumin, total protein, INR
    - 10ml Heparin blood
- HCV-RNA
    - 10ml EDTA blood
- Drug level of current ART
    - 10ml EDTA blood
    - Note the time-point (date, hour, minutes) of the last intake and the dosage (milligram) on the electronic CRF
    - Blood (10 ml) must be collected in EDTA solution vacutainer tubes. The tubes containing EDTA solution should be stored at +4°C. Tubes must be identified with the patient ID number and sample number as soon as blood has been collected
    - Blood samples must be brought to the laboratories without delay (maximum 30 min after blood collection).
    - Treatment of samples: centrifugation at 1500g for 10 min at +4°C. In a class II biohazard hood plasma (obtained after centrifugation) should be divided in three labelled tubes (of approximately 1mL each) ready for freezing at -80 ° C, within one hour after collection. If this deadline cannot be met, please keep the sample at 4° C until centrifugation and freezing
- Silibinin drug-level (Cmin (T0, pre-dose), Cmax (i.e immediately after the end of silibinin infusion) and one sampling at T4 (i.e. 4 hour after the end of silibinin infusion)
    - Blood (6 ml) must be collected in ACD (acid citrate dextrose) solution vacutainer tubes. The tubes containing ACD solution should be stored at +4°C. Tubes must be identified with the patient ID number and sample number as soon as blood has been collected
    - Blood samples must be brought to the laboratories without delay (maximum 30 min after blood collection).
    - Treatment of samples: centrifugation at 1500g for 10 min at +4°C. In a class II biohazard hood plasma (obtained after centrifugation) should be divided in three labelled tubes (of approximately 1mL each) ready for freezing at -80 ° C, within one hour after collection. If this deadline cannot be met, please keep the sample at 4° C until centrifugation and freezing.
- Plasma aliquot as backup-blood for potential later analysis

    - 10ml ETDA blood
- PBMC: only at the study site Zurich
    - 10ml Heparin blood
- Administration of iSIL 20mg/Kg during 1 hour
    - Preparation of solution see below in the protocol
    - Vital parameters before, every 15min and after administration of iSIL
    - Documentation of side effects in the electronic CRF
- Accepted time window: same day

9.14 Day 13 (visit 14)

- Documentation of adverse events
- Concomitant medication
- HCV-RNA,
    - 10ml EDTA blood
- Administration of iSIL 20mg/Kg during 1 hour
    - Preparation of solution see below in the protocol
    - Vital parameters before, every 15min and after administration of iSIL
    - Documentation of side effects in the electronic CRF
- Accepted time window: same day

9.15 Day 14 (visit 15)

- Documentation of adverse events
- Concomitant medication
- HCV-RNA,
    - 10ml EDTA blood
- Administration of iSIL 20mg/Kg during 1 hour
    - Preparation of solution see below in the protocol
    - Vital parameters before, every 15min and after administration of iSIL
    - Documentation of side effects in the electronic CRF
- Accepted time window: same day

9.16 Day 15 (visit 16, end of THISTLE)

- Documentation of adverse events
  - use the electronic CRF
- Concomitant medication
  - use the electronic CRF
- Physical examination
  - Including blood pressure while sitting (right/left), pulse rate, temperature (degree Celsius), cardiac auscultation, chest auscultation, abdominal auscultation, brief neurological examination
- Safety laboratory: full blood count, electrolytes (sodium, potassium), ASAT, ALAT, total and fractionated bilirubin, gamma GT, alcaline phosphatase, creatinine, albumin, total protein, INR
  - 10ml Heparin blood
- Pregnancy test:
  - 10ml Heparin blood
- HCV-RNA, HIV-RNA
  - 10ml EDTA blood
- CD3/4/8 count
  - 3ml EDTA blood
- Plasma aliquot as backup-blood for potential later analysis
  - 10ml ETDA blood
- PBMC: only at the study site Zurich
- Silibinin drug-level T24 (i.e 24 hour after the end of last silibinin infusion)
  - Blood (6 ml) must be collected in ACD (acid citrate dextrose) solution vacutainer tubes. The tubes containing ACD solution should be stored at +4°C. Tubes must be identified with the patient ID number and sample number as soon as blood has been collected
  - Blood samples must be brought to the laboratories without delay (maximum 30 min after blood collection).
  - Treatment of samples: centrifugation at 1500g for 10 min at +4°C. In a class II biohazard hood plasma (obtained after centrifugation) should be divided in three labelled tubes (of approximately 1mL each) ready for freezing at -80 ° C, within one hour after collection. If this deadline cannot be met, please keep the sample at 4° C until centrifugation and freezing.
- Accepted time window: at least one day after the end of iSIL administration

Figure 2: Flow-chart with timing of the assessments

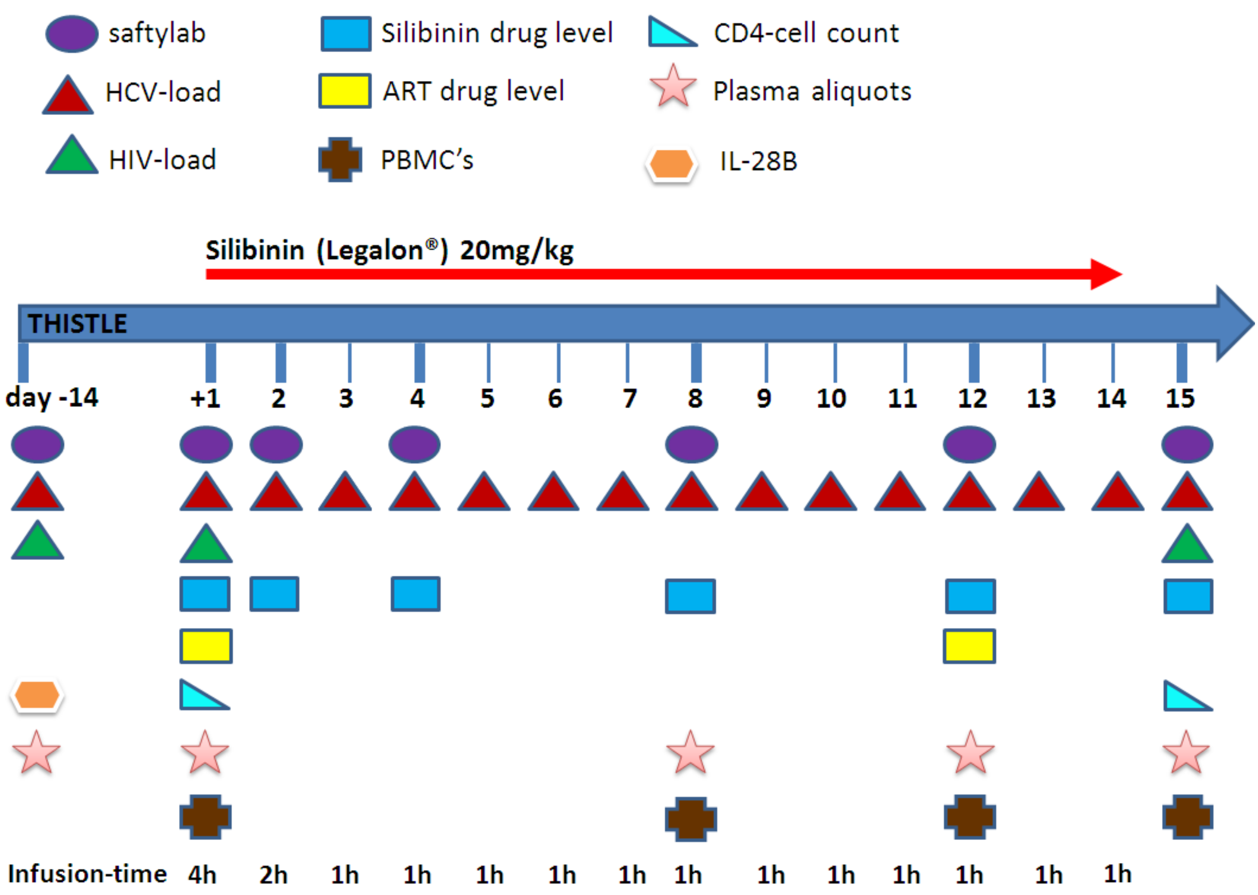

9.17 Sample preparation, storage, and shipment

All tubes and labels will be provided for each sample by the lab of the study-site. Each sample storage tube label should include protocol number, patient number, visit number and drug name. The date and actual clock time of each sample collection will be accurately recorded in the eCRF.

<u>HIV-1 RNA, HCV-RNA, CD4 cells</u>

- The blood samples will be stored for five years and then will be destroyed definitively.

<u>Silibinin PK:</u>

- Blood (6 ml) must be collected in ACD (acid citrate dextrose) solution vacutainer tubes. The tubes containing ACD solution should be stored at +4°C. Tubes must be identified with the patient ID number and sample number as soon as blood has been collected

- Blood samples must be brought to the laboratories without delay (maximum 30 min after blood collection).
- Treatment of samples: centrifugation at 1500g for 10 min at +4°C. In a class II biohazard hood plasma (obtained after centrifugation) should be divided in three labelled tubes (of approximately 1mL each) ready for freezing plasma at -80 ° C, within one hour after collection. If this deadline cannot be met, please keep the sample at 4° C until centrifugation and freezing.
- The blood samples will be stored for 10 years and then will be destroyed definitively.

<u>Drug level of antiretroviral drugs:</u>

- Blood (10 ml) must be collected in EDTA solution vacutainer tubes. The tubes containing EDTA solution should be stored at +4°C. Tubes must be identified with the patient ID number and sample number as soon as blood has been collected
- Blood samples must be brought to the laboratories without delay (maximum 30 min after blood collection).
- Treatment of samples: centrifugation at 1500g for 10 min at +4°C. In a class II biohazard hood plasma (obtained after centrifugation) should be divided in three labelled tubes (of approximately 1mL each) ready for freezing plasma at -80 ° C, within one hour after collection. If this deadline cannot be met, please keep the sample at 4° C until centrifugation and freezing.
- The blood samples will be stored for 10 years and then will be destroyed definitively.

<u>Plasma aliquots:</u>

- Two aliquots of EDTA plasma samples will be obtained at each study site on the screening visit, visit 1, 8, 12, 15. The aliquots will be used as backup-blood for potential later analysis. The aliquots should contain at least 5 mL plasma. The plasma samples will be stored at -20°C or colder.
- The plasma aliquots will be stored for 5 years in accordance to the biobank-protocol if a separate informed consent form was obtained.

<u>PBMC's:</u>

- PBMC's will only be obtained at the study site in Zurich
- One PBMC sample will be obtained on visit 1, 12 and 15. The PBMC sample should contain at least 10 mL heparin blood. The samples will be stored at -20°C or colder
- The PBMC's will be stored for 5 years in accordance to the biobank-protocol if a separate informed consent form was obtained.

The PK plasma and aliquots will be shipped to the central laboratory in Lausanne (laboratory Prof. Laurent Decosterd). Samples must be shipped with adequate dry ice in a suitable, durable shipment container to assure that samples remain frozen during transit. Enclose the inventory of samples with each shipment. The central lab will then check the PK samples to ensure the planned samples are accounted for and can be

properly identified. At the end of the study, one part of the stored plasma for PK-measures will be sent from the laboratory in Lausanne to RPH (Monza) RPH will also perform PK-determinations with its own developed assay. This procedure allows us to perform a “cross-over” validation of the different assays for drug-level measures.

The duration of sample storage will follow the respective SAMW Guideline regarding the collection of human biological material (Biobanking)

10 SAFETY

10.1 Definition of (Serious) Adverse Events

Adverse events

Adverse events (AEs) are defined as any untoward medical occurrence in a patient or clinical investigation subject administered a pharmaceutical product and which does not necessarily have a causal relationship with this treatment.

An AE can therefore be any unfavourable and unintended sign (including an abnormal laboratory finding), symptom, or disease temporally associated with the use of a medicinal study product, whether or not related to the medicinal study product. An AE may also consist of a new disease, an exacerbation of a pre-existing illness or condition, a recurrence of an intermittent illness or condition, a set of related signs or symptoms, or a single sign or symptom. The definition covers also medication errors and uses outside what is foreseen in the protocol, including misuse and abuse of the product, as well as events that could be associated with study procedures and which could modify the conduct of the trial.

AEs observed by the investigator and/or reported by the subject must be reported in the eCRF during the entire study period, i.e. the period of time from the first (= signature of informed consent) to the last protocol-specific procedure regardless of the medicinal study product relation assessment.

For all AEs, sufficient information will be pursued and/or obtained so as to permit an adequate determination of the outcome of the event (i.e., whether the event should be classified as an SAE) and an assessment of the casual relationship between the AE and the investigational drug or study treatment(s).

Whenever available, the underlying disease or condition for which a therapeutic or diagnostic procedure is required should be reported as the AE term. Surgeries or other invasive procedures that had already been planned prior to the start of the study do not have to be documented as AEs. These planned procedures will be recorded in the eCRF by the investigator at the baseline visit. It is not important if the condition was known before enrolment, only if the procedure was planned before.

Pregnancy per se does not classify as an AE. However, AEs related to a pregnancy have to be reported like any other AEs. Pregnancy should be confirmed by a reliable laboratory test. Pregnant subjects must be immediately withdrawn from the clinical study. All pregnancies occurring during the treatment phase of the study and within 30 days after discontinuation of study medication have to be reported to the Investigator-Sponsor within 24h of the investigational sites knowledge of the pregnancy on the Initial Pregnancy Report Form. The Sponsor-Investigator will contact the attendant physician by phone during pregnancy and after the estimated date of delivery to enquire about course and outcome of the pregnancy. Course of the pregnancy and health status of the new born child have to be documented on the Follow-Up Pregnancy Report Form.

Serious adverse event

An SAE is any untoward medical occurrence that at any dose results in

- results in death,
- is life-threatening,
- requires subject hospitalization or prolongation of current hospitalization,
- results in persistent or significant disability/incapacity, or
- is a congenital anomaly/birth defect,
- any important medical event and any event which, though not included in the above, may jeopardise the subject or may require intervention to prevent one of the outcomes listed above.

Any other medically important condition that may be not immediately life-threatening or results in death or hospitalization but may jeopardize the subject or may require intervention to prevent one of the outcomes listed above should also usually (i.e. based on medical and scientific judgment) be considered serious. For example: intensive treatment at home for allergic bronchospasm; certain laboratory abnormalities (e.g. blood dyscrasias); convulsions that do not result in hospitalisation; development of drug dependency or drug abuse.

Suspected unexpected serious adverse reaction (SUSAR)
A serious adverse reaction, the nature or severity of which is suspected to be not consistent with the applicable product information (current version of Legalon® SIL Investigator's Brochure, see section 10.6 for details).

First Awareness Date (FAD)
The first date any SPONSOR's employee or designee becomes aware of the minimum information for considering a case valid (i.e. (i) an identifiable patient, (ii) a suspect product, (iii) an identifiable reporting source, and (iv) an event). The clock for reporting purposes starts at the date of initial receipt (FAD) and it is defined as day 0.

Adverse Drug Reactions (ADRs)
"All untoward and unintended responses to an investigational medicinal product related to any dose administered".

10.2 Recording of (Serious) Adverse Events

Clinical study subjects will be routinely questioned about AEs at study visits. The well-being of the subjects will be ascertained by neutral questioning ("How are you?"). The investigator is responsible for reporting all AEs occurring during the course of the study.

All observed or volunteered adverse drug events (serious or non-serious) and abnormal test findings, regardless of treatment group or suspected causal relationship to the investigational drug or study treatment(s) will be recorded in the patient file and subsequently in the eCRF.

AEs or abnormal test findings felt to be associated with the study treatment(s) will be followed until the event (or its sequelae) or the abnormal test finding resolves or stabilizes at a level acceptable to the investigator.

An abnormal test finding will be classified as an AE if one or more of the following criteria are met:

- The test finding is accompanied by clinical symptoms.
- The test finding necessitates additional diagnostic evaluation(s) or medical/surgical intervention; including significant additional concomitant drug treatment or other therapy

  Note: simply repeating a test finding, in the absence of any of the other listed criteria, does not constitute an AE.
- The test finding leads to a change in study dosing or discontinuation of subject participation in the clinical study.

All AEs, serious and non-serious, will be fully documented in the appropriate eCRF. For each AE, the investigator will provide the onset, duration, intensity, treatment required, outcome and action taken with the investigational product.

The intensity of AEs will be assessed as being

- mild (hardly noticeable, negligible impairment of well-being),
- moderate (marked discomfort, but tolerable without immediate relief), or
- severe (overwhelming discomfort, calling for immediate relief).

The investigator will determine the relationship of the investigational drug to all AEs as defined on the AE page of the eCRF.

10.3 Assessment of (Serious) Adverse Events

The investigator will promptly review documented AEs and abnormal test findings to determine if

- the abnormal test finding should be classified as an AE,
- if there is a reasonable possibility that the AE was caused by the investigational drug or study treatment(s), and
- if the AE meets the criteria for an SAE.

The assessment by the investigator with regard to the study drug relation is done according to the following definitions:

<u>No reasonable causal relationship</u>
There is no reasonable causal relationship between the administration of the study drug and the event.

- no temporal relationship to drug administration
- other drugs or chemicals or underlying disease provides definite explanations

Reasonable causal relationship
There is a reasonable causal relationship between the administration of the study drug and the event. The time sequence and the evaluation of other elements of the AE show that there is a possible causal relationship with the study drug.
Reasonable causal relationship may be further defined as:

Possible

An AE, which

- occurs within a reasonable time sequence to administration of the drug
- but could also be explained by concurrent disease or other drugs or chemicals

Probable

An AE, which

- occurs within a reasonable time sequence to administration of the drug
- is unlikely to be attributed to concurrent disease or other drugs or chemicals

Definitely

An AE, which

- plausible time relationship to IMP administration
- cannot be explained by concurrent disease or other drugs or chemicals

10.4 Reporting of Serious Adverse Events

The Principal Investigator from each study site is responsible for SAE reporting to the Sponsor according to the following details:

- Reporting to the Sponsor any SAE:

  - **without delay within 24hours** by FAX (044 255 32 91), E-Mail (jan.fehr@usz.ch) or during the weekend (Saturday and Sunday) by Mobile-Phone (+41 79 677 28 77)

- Reporting to local IEC of non-fatal and not life-threatening SAEs if evaluated as “suspected”, “unexpected” and “drug related” (SUSAR):
- **- promptly** and no later than **15 calendar days** following awareness that event meets criteria for a SUSAR by FAX or E-Mail as specified by the local IEC. Reporting to local IEC of fatal and life-threatening SAEs if evaluated as ”drug related” (SADR) or “suspected”, “unexpected” and “drug related” (SUSAR):

  - **without delay** and no later than **7 calendar days** following awareness that event meets criteria for a SUSAR by FAX or E-Mail as specified by the local IEC.

  - follow-up information regarding the SUSAR within **further 8 calendar days**.

- All other SAEs will be summed up in the **annual safety update report**.

The Sponsor is responsible for SUSAR reporting to Swissmedic according to the following details:

- Reporting to Swissmedic of fatal and life-threatening SAEs if evaluated as “suspected”, “unexpected” and “drug related” (SUSAR):

  - **without delay** and no later than **7 calendar days** following awareness that event meets criteria for a SUSAR;

  - follow-up information regarding the SUSAR within **further 8 calendar days**.

- Reporting to Swissmedic of non-fatal and not life-threatening SAEs if evaluated as “suspected”, “unexpected” and “drug related” (SUSARs):

  - **promptly** and no later than **15 calendar days** following awareness that event meets criteria for a SUSAR.

- Sending yearly safety reports, starting one year after the date of notification to Swissmedic. The reports will be drafted by the local Principal Investigator of each study site and sent to the Sponsor. These reports should contain:
- - A concise critical summary of the safety profile of the drug studied as well as the safety issues that have arisen;
- - A listing of all SUSARs that have occurred -The accompanying letter provided with the Annual Safety Report should contain a short summary of the status of the clinical trial in Switzerland (number of centres open/closed, number of patients recruited/recruitment closed, and number of SAR/SUSAR. Reporting immediately all suspected new risks and relevant aspects of known unexpected reactions that require safety-related measure.

An unexpected SAE refers to any AE, the nature or severity of which is not consistent with the current version of Legalon® SIL Investigator’s Brochure (see section 10.6 for details).

10.5 Follow up of (Serious) Adverse Events

Subjects terminating the study (either regularly or prematurely) with

- reported ongoing SAE, or
- any ongoing AEs of laboratory values or of vital signs being beyond the alert limit

will return for a follow-up investigation. This visit will take place up to 30 days after terminating the treatment period. Follow-up information on the outcome will be recorded on the respective AE page in the eCRF. All other information has to be documented in the source documents. Source data has to be available upon request.

In case of subjects lost to follow-up, efforts should be made and documented to contact the subject to encourage him/her to continue study participation as scheduled. In case of minor AEs a telephone call to the subject may be acceptable.

All new SAE or pregnancies that the investigators will be notified of within 30 days after discontinuation of study medication have to be reported in appropriate report forms and in the eCRF if required.

Follow-up investigations may also be necessary according to the investigator's medical judgment even if the subject has no AE at the end of the study. However, information related to these investigations does not have to be documented in the eCRF but must be noted in the source documents.

10.6 Potential risks/adverse events and treatment

Due the limited size of the patient population with HCV treated with Legalon® SIL, the estimated incidence of possible adverse reactions provided below is based on the balance between the uncertainty of the actual proportions observed and the low absolute number of patients reporting the reaction, in comparison with the control group in controlled studies and taking into account the supposed relationship with treatment:

- Very common: sensation of heat.
- Common: diarrhoea, bilirubin increased, nausea, vomiting, headache.
- Uncommon: abdominal pain, fever, asthenia, shivering, feeling of body temperature change, abdominal discomfort.
- Rare: somnolence.

It should also be considered that the above incidences are not readily transferable to the general HCV patient population (probably with the exception of sensation of heat, diarrhoea and bilirubin increased), as most of the data on which they are based were derived from studies in special patient subgroups, often in critical conditions, such as patients in liver peri- or post-transplant situations.

Sensation of heat, feeling of body temperature change, shivering and fever usually occur during infusions at the beginning of treatment with Legalon® SIL and recede spontaneously on continuation of therapy. Slow infusions of Legalon® SIL might reduce their occurrence.

Bilirubin increase is a transient effect which spontaneously subsides upon Legalon® SIL discontinuation without treatment. Data collected so far indicate that bilirubin increase following Legalon® SIL administration is not accompanied by cholestatic or hepatotoxic effects.

No contra-indications are known other than hypersensitivity to the active substance or to the excipients. No interactions with other medicine or other forms of interaction are known.

The risk for clinically relevant metabolic drug interactions can be considered low based on in vitro studies and supported by clinical data collected in patients receiving Legalon® SIL and other concomitant medications who did not require any dose adjustments.

There are no adequate data from the use of Legalon® SIL in pregnant women and for this reason Legalon® SIL administration during pregnancy and lactation is not recommended.

There are no reported effects on ability to drive and use machines.
No case of overdose has been reported.

Legalon® SIL is to be administered by intravenous infusions. Thus patients will be exposed to the general risks related to prolonged and repeated catheter insertion. These risks might be mitigated following the "Guidelines for the Prevention of Intravascular Catheter-Related Infections" (20). The Investigators are recommended to strictly adhere to these guidelines.

10.7 Assessment of safety laboratory parameters

The laboratory tests listed in Table below will be performed at the laboratory of each study site and the laboratory of Prof. Decosterd in Lausanne. For time points of laboratory sampling refer to the Flow Chart. Laboratory results of the patients will be available to the investigator of each study site and selected abnormal laboratory alerts will be sent automatically to the sponsor within 24 hours. Clinically relevant laboratory values should be commented on lab report print-outs. A clinically relevant value may be either in- or outside the reference range. Out-of-range data judged as clinically significant (CS) by the Investigator has to be report as an adverse event. Clinically relevant abnormal laboratory test results must be confirmed and should be repeated until normalization or stabilization or until an alternative explanation has been found.

Category	Test name
Haematology	Hematocrit (Hct)<br>Hemoglobin (Hb)<br>Red Blood Cell Count/ Erythrocytes<br>White Blood Cells / Leukocytes<br>Platelet Count/ Thrombocytes<br>Diff. Automatic Neutrophils (relative count)<br>Eosinophils (relative count)<br>Basophils (relative count)<br>Monocytes (relative count)<br>Lymphocytes (relative count)
Coagulation	Prothrombin time (Quick and INR)
Chemistry	AST(GOT)<br>ALT(GPT)<br>Gamma GT<br>Albumin<br>Total protein<br>Alkaline Phosphatase (AP)<br>Sodium<br>Potassium<br>Glucose<br>Creatinine and creatinine clearance*<br>Bilirubin Total and fractionated
Lymphocyte subset	CD3+ Lymphocyte (absolute and %)<br>CD4+ Lymphocyte (absolute and %)<br>CD8+ Lymphocyte (absolute and %)
Serum Pregnancy test	Human Serum chorionic gonadotropin
Hormones	TSH
HCV	HCV genotyping assay PCR/LIPA<br>Plasma HCV RNA (quantitative) with Roche COBAS TaqMan v1.0 sensitivity range <15 UI/ml

HIV	Plasma HIV RNA (quantitative) Roche COBAS TaqMan v2.0 sensitivity range <20
Drug level	Concurrent ART drug levels<br>Silibinin drug level

10.8 Pregnancy test

Serum pregnancy tests will be performed at screening (Visit 1) and at the final visit (visit 7) for all female patients of childbearing potential.

10.9 Physical examination

Complete and target physical examinations will be carried out as described at the Flow Chart. A complete physical exam consists of an evaluation of organ systems with vital signs (including temperature, pulse rate, and systolic/diastolic blood pressure).

Respiratory rate, temperature, pulse rate, and blood pressure will be measured after patients have been sitting comfortably for at least two minutes.

A targeted physical examination includes an evaluation of vital signs and organ systems only associated with adverse event symptoms as needed, and particularly focused on hepatic related events.

Clinically relevant abnormal findings will be reported as adverse events.

10.10 Exchange of safety data between sponsor and Rottapharm

The drug safety data and the information regarding the study treatment will be exchanged between Rottapharm Madaus and the Sponsor in accordance to the pharmacovigilance agreement (we refer to the separate document “pharmacovigilance”). This exchange is aimed to ensure both Parties have the necessary information to monitor and evaluate the study treatment safety profile and to fulfill reporting requirements in the territories under their responsibility. The Parties exchange information utilizing the most current version of Medical Dictionary for Regulatory Activities (MedDRA) as recommended by the Maintenance and Support Service Organization (MSSO). Additionally, each Party code, when possible, all suspect and concomitant drugs with the World Health Organization’s, WHO Drug Dictionary (WHO DRUG).

11 DATA QUALITY ASSURANCE

The Sponsor-Investigator is implementing and maintaining quality assurance and quality control systems with written SOPs and Working Instructions to ensure that trials are conducted and data are generated, documented (record), and reported in compliance with the protocol, GCP, and applicable regulatory requirement(s).

Monitoring will be conducted during the course of the study for quality assurance purposes.

Since almost all study-subjects are included in the SHCS, which is internationally recognized for its high and the well-established and maintained structure (www.shcs.ch), an ongoing data-cleaning is warranted. We therefore not plan to perform primary an audit during the study.

11.1 Routine Monitoring

Regular monitoring visits at the investigator's site prior to the start and during the course of the study will help to follow up the progress of the clinical study, to assure utmost accuracy of the data and to detect possible errors at an early time point.

To achieve this goal, the monitor will carry out the following visits:

- Trial Initiation Monitoring Visit aimed at ensuring the Principal Investigator (PI) and the Staff are properly instructed on how to follow the study protocol and to conduct the trial; this assumes all required essential documents for the trial have been obtained and the IMPs are available at Site
- Monitoring Visits aimed at verifying that the rights and well-being of human subjects are protected; the reported trail data are accurate, complete, and verifiable from source documents; the conduct of the trial is in compliance with the currently approved protocol/amendments, with ICH-GCP, and with all the applicable regulatory requirements.
- Close-out Monitoring Visit aimed at ensuring essential documents are available; arrangements for retention of study and source data have been made; all remaining unused supplies are returned and/or destroyed.

The Sponsor-Investigator organises professional independent monitoring for the study.

All original data including all patient files, progress notes and copies of laboratory and medical test results must be available for monitoring. The monitor will review all or a part of the eCRFs and written informed consents. The accuracy of the data will be verified by reviewing the above referenced documents. The investigator's site will collaborate with the Clinical Trials Center (CTC) of the University Hospital Zurich to ensure regular monitoring. According to the CTC's Monitoring SOP the extent and nature of monitoring activities based on the objective and design of the study will be defined in a study specific Monitoring Plan.

11.2 Audits and Inspections

A quality assurance audit/inspection of this study may be conducted by the regulatory authority or IEC, respectively. The quality assurance auditor/inspector will have access

to all medical records, the investigator's study related files and correspondence, and the informed consent documentation that is relevant to this clinical study.

The investigator will allow the persons being responsible for the audit or the inspection to have access to the source data/documents and to answer any questions arising. All involved parties will keep the patient data strictly confidential.

11.3 Specification of Source Documents

In addition to the medical chart of the patients and the laboratory reports, the following documents are considered source data, including but not limited to:

• SAE worksheets

• Nurse records, records of clinical coordinators, and

• Medical records from other department(s), or other hospital(s), or discharge letters and correspondence with other departments/hospitals, if subject visited any during the study period and the post study period.

Source data must be available at the site to document the existence of the study subjects and substantiate the integrity of study data collected. Source data must include the original documents relating to the study, as well as the medical treatment and medical history of the subject.

The following information (at least but not limited to) should be included in the source documents:

• Demographic data (age, sex)

• Inclusion and Exclusion Criteria details

• Participation in study and signed and dated Informed Consent Forms

• Visit dates

• Medical history, prior concomitant medication and physical examination details

• Key efficacy and safety data (as specified in the protocol)

• AEs and concomitant medication

• Results of relevant examinations

• Laboratory printouts

• Dispensing and return of study drug details

• Reason for premature discontinuation

• Patient ID number

12 STATISTICS

12.1 Statistical and Analytical Plans

The analyses of the primary and secondary endpoints of this non-comparative study will be purely descriptive. The average decline of HCV RNA during the lead-in phase (day +1 to day +15) will be expressed in log_{10} IU/ml using methods for left-censored data (Tobit regression) (18) in case of undetectable values are encountered and represents the primary efficacy analysis and will be performed in the evaluable patients. We will further report the percentages (with 95% CI) of patients achieving undetectable levels of HCV RNA at the various time points. We will also attempt to analyse factors associated with log_{10} HCV RNA response with mixed effects models (19) taking into account all measured HCV values from day -14 to day + 15. In these models, slopes will be allowed to change at day+1 and day +15. Potential co-factors will be first assessed in univariable and bivariable models to detect interactions and collinearities. Relevant effect modifiers and confounders will then be included in multivariable models. The following co-factors will be considered: age, gender, IL28B genotype, BMI, CD4 values and HIV RNA. We will look at the potential co-factors in univariable models first and only include relevant confounding variables in the multivariable analysis. Safety analyses will be performed including all patients that received at least one dose of the study medication (Safety Population) Adverse events will be reported in percentages (with 95% CI) of patients experiencing an event (for clinical events and abnormal laboratory values considered clinically significant by the Investigator – this represent the primary safety analyses) or the percentages below/above 1x and 2x normal range limits for laboratory values.

12.2 Null and Alternative Hypotheses

Not applicable.

12.2.1 Interim Analysis

There will be no interim analysis in this small study.

12.2.2 Deviation(s) from the original statistical plan

Not applicable.

12.3 Handling of Missing Data

We plan to recruit additional patients in case of drop-outs or extensive missing data to ascertain that there are 20 evaluable patients. We will further evaluate the effect of missing data on study findings by three sensitivity analyses (missing=failure, missing=success, last observation carried forward).

12.4 Determination of Sample Size

In concordance to common safety- or PK-studies a number of 20 enrolled study-subjects should be adequate to make a clear statement regarding safety-issues. During the short lead-in course of 14-days iSIL treatment we expect no significant confounders which significantly influence the primary and secondary outcomes. Because the preliminary data show such a strong effect of iSIL regarding the HCV-RNA decline from

baseline, we expect to demonstrate a clear efficacy of iSIL with a sample size of 20 subjects.

13 DATA HANDLING AND RECORD KEEPING

The study will strictly follow the protocol. If any changes become necessary, they must be laid down in an amendment to the protocol. All amendments of the protocol must be signed by the Sponsor-Investigator and submitted to IEC and Swissmedic.

The investigators will use electronic case report forms (eCRF), one for each enrolled study participant, to be filled in with all relevant data pertaining to the subject during the study. All subjects who either entered the study or were considered not-eligible or were eligible but not enrolled into the study additionally have to be documented on a screening log. The investigator will document the participation of each study subject on the enrolment Log.

For data and query management, monitoring, reporting and coding an internet-based secure data base SecuTrial® developed in agreement to the Good Clinical Practice (GCP) guidelines provided by the Clinical Trials Center (CTC) Zurich will be used for this study. It is the responsibility of the investigator to assure that all data in the course of the study will be entered completely and correctly in the respective data base. Corrections in the eCRF may only be done by the investigator or by other authorised persons. In case of corrections the original data entries will be archived in the system and can be made visible. For all data entries and corrections date, time of day and person who is performing the entries will be generated automatically.

eCRFs must be kept current to reflect subject status at each phase during the course of study. Subjects must not to be identified in the eCRF by name. Appropriate coded identification (e.g. Subject Number) must be used.

It must be assured that any authorised person, who may perform data entries and changes in the eCRF, can be identified. A list with signatures and initials of all authorised persons will be filed in the study site file and the trial master file, respectively.

Documented medical histories and narrative statements relative to the subject's progress during the study will be maintained. These records will also include the following: originals or copies of laboratory and other medical test results (e.g. ECGs, etc.) which must be kept on file with the individual subject's eCRF.

The investigators assure to perform a complete and accurate documentation of the subject data in the source documents. All data entered into the eCRF must also be available in the individual subject file either as print-outs or as notes taken by either the investigator or another responsible person assigned by the investigator.

Essential documents must be retained for at least 10 years after the regular end or a premature termination of the respective study (VKlin Art. 25).

Any patient files and source data must be archived for the longest possible period of time according to the feasibility of the investigational site, e.g. hospital, institution or private practice.

14 CONFIDENTIALITY

The investigators are liable to treat the entire information related to the study and the compiled data strictly confidentially. Any passing-on of information to persons that are not directly involved in the study must be approved by the owner of the information.

Data generation, transmission, archiving and analysis of personal data within this study, strictly follows the current Swiss legal requirements for data protection. Prerequisite is the voluntary approval of the subject given by signing the informed consent prior start of participation of the clinical trial.

Individual subject medical information obtained as a result of this study is considered confidential and disclosure to third parties is prohibited. Subject confidentiality will be further ensured by utilising subject identification code numbers to correspond to treatment data in the computer files.

Such medical information may be given to the subject's personal physician or to other appropriate medical personnel responsible for the subject's welfare, if the patient has given his/her written consent to do so.

Data generated as a result of this study are to be available for inspection on request by the Sponsor, by the IEC and the regulatory health authorities.

15 INSURANCE

Insurance is covered by “Haftpflichtversicherung für den Kanton Zürich betreffend das UniversitätsSpital Zürich“.

Any damage developed in relation to study participation is covered by this insurance. So as not to forfeit their insurance cover, the subjects themselves must strictly follow the instructions of the study personnel. Subjects must not be involved in any other medical treatment without permission of the principal investigator (emergency excluded). Medical emergency treatment must be reported immediately to the investigator. The investigator must also be informed instantly, in the event of health problems or other damages during or after the course of study treatment.

The investigator will allow delegates of the insurance company to have access to the source data/documents as necessary to clarify a case of damage related to study participation. All involved parties will keep the patient data strictly confidential.

A copy of the insurance certificate will be placed in the Investigator’s Site File.

16 STUDY REGISTRATION

The study will be registered in the local trial registry of the University Hospital Zürich („Studienregister USZ“) and in an international trial registry, e.g. ClinicalTrials.gov (clinicaltrials.gov).

17 PUBLICATION POLICY

After the statistical analysis of this trial the sponsor will make every endeavour to publish the data in a medical journal.

18 SIGNATURES

Sponsor:

This clinical trial protocol was subject to critical review and has been approved by the Sponsor-Investigator. The information herein is consistent with

- the current risk/benefit evaluation of the investigational product(s)
- the moral, ethical and scientific principles governing clinical research as set out in the current version of the Declaration of Helsinki, Good Clinical Practice and the respective SAMW guidelines.

Jan Sven Fehr

Zurich,

Place/Date	Signature

Principal Investigators:

I have read and understood this trial protocol and agree to conduct the trial as set out in this study protocol.

Dominique Laurent Braun

Zurich,

Place/Date	Signature

Andri Rauch

Berne,

Place/Date	Signature

Marcel Stöckle

Basel,

Place/Date | Signature

Juan Ambrosioni

Geneva,

Place/Date | Signature

Matthias Cavassini

Lausanne,

Place/Date | Signature

Patrick Schmid

Sankt Gallen,

Place/Date | Signature

Enos Bernasconi

Lugano,

Place/Date | Signature

Biometrician:

Bruno Ledergerber

Place/Date | Signature

REFERENCES

1. Perz JF, Armstrong GL, Farrington LA, Hutin YJ, Bell BP. The contributions of hepatitis B virus and hepatitis C virus infections to cirrhosis and primary liver cancer worldwide. J Hepatol. 2006 Oct;45(4):529-38. PubMed PMID: 16879891. eng.
2. Andrea W, Patrick S. Hepatitis C update 2010. Swiss Medical Forum2010. p. 729-36.
3. Rauch A, Rickenbach M, Weber R, Hirschel B, Tarr PE, Bucher HC, et al. Unsafe sex and increased incidence of hepatitis C virus infection among HIV-infected men who have sex with men: the Swiss HIV Cohort Study. Clin Infect Dis. 2005 Aug;41(3):395-402. PubMed PMID: 16007539. eng.
4. Bundesamt für Gesundheit Zahlen und Fakten zur Lebertransplantation 2010.
5. Ingiliz P, Rockstroh JK. HIV-HCV co-infection facing HCV protease inhibitor licensing: implications for clinicians. Liver Int. 2012 Apr. PubMed PMID: 22510096. ENG.
6. Sulkowski MS. Hepatitis C virus-human immunodeficiency virus coinfection. Liver Int. 2012 Feb;32 Suppl 1:129-34. PubMed PMID: 22212583. eng.
7. Dietrich D, Sulkowski M. Telaprevir in Combination with Pegylated Interferon-a-2a+RBV in HCV/HIV-co-infected Patients: A 24-Week Treatment Interim Analysis. CROI2012.
8. Wagoner J, Morishima C, Graf TN, Oberlies NH, Teissier E, Pécheur EI, et al. Differential in vitro effects of intravenous versus oral formulations of silibinin on the HCV life cycle and inflammation. PLoS One. 2011;6(1):e16464. PubMed PMID: 21297992. Pubmed Central PMCID: PMC3030583. eng.
9. Ahmed-Belkacem A, Ahnou N, Barbotte L, Wychowski C, Pallier C, Brillet R, et al. Silibinin and related compounds are direct inhibitors of hepatitis C virus RNA-dependent RNA polymerase. Gastroenterology. 2010 Mar;138(3):1112-22. PubMed PMID: 19962982. eng.
10. Guedj J, Dahari H, Pohl RT, Ferenci P, Perelson AS. Understanding silibinin's modes of action against HCV using viral kinetic modeling. J Hepatol. 2012 May;56(5):1019-24. PubMed PMID: 22245888. Pubmed Central PMCID: PMC3328661. eng.
11. Payer BA, Reiberger T, Rutter K, Beinhardt S, Staettermayer AF, Peck-Radosavljevic M, et al. Successful HCV eradication and inhibition of HIV replication by intravenous silibinin in an HIV-HCV coinfected patient. J Clin Virol. 2010 Oct;49(2):131-3. PubMed PMID: 20709593. eng.
12. Rutter K, Scherzer TM, Beinhardt S, Kerschner H, Stättermayer AF, Hofer H, et al. Intravenous silibinin as 'rescue treatment' for on-treatment non-responders to pegylated interferon/ribavirin combination therapy. Antivir Ther. 2011;16(8):1327-33. PubMed PMID: 22155914. eng.
13. Biermer M, Thomas B. High-dose silibinin rescue treatment for HCV-infected patients showing suboptimal virological response to standard combination therapy. Journal of Viral Hepatitis. 2011.
14. Eurich D, Bahra M, Berg T, Boas-Knoop S, Biermer M, Neuhaus R, et al. Treatment of hepatitis C-virus-reinfection after liver transplant with silibinin in nonresponders to pegylated interferon-based therapy. Exp Clin Transplant. 2011 Feb;9(1):1-6. PubMed PMID: 21605016. eng.

15. Moreno A, Moreno S. Safety and Anti-HCV Activity of Rescue Therapy with Intravenous Silibinin in Cirrhotic HIV/HCV-Co-infected Subjects. CROI2012.
16. Ferenci P, Scherzer TM, Kerschner H, Rutter K, Beinhardt S, Hofer H, et al. Silibinin is a potent antiviral agent in patients with chronic hepatitis C not responding to pegylated interferon/ribavirin therapy. Gastroenterology. 2008 Nov;135(5):1561-7. PubMed PMID: 18771667. eng.
17. Castera L, Forns X, Alberti A. Non-invasive evaluation of liver fibrosis using transient elastography. J Hepatol. 2008 May;48(5):835-47. PubMed PMID: 18334275. eng.
18. Econometrica1958. 24-6 p.
19. Chu H, Gange SJ, Li X, Hoover DR, Liu C, Chmiel JS, et al. The effect of HAART on HIV RNA trajectory among treatment-naïve men and women: a segmental Bernoulli/lognormal random effects model with left censoring. Epidemiology. 2010 Jul;21 Suppl 4:S25-34. PubMed PMID: 20386106. eng.
20. CDC Atlanta, Guidelines for the Prevention of Intravascular Catheter-Related Infections, 2011_O'Grady Naomi P.; also available at http://www.cdc.gov/hicpac/BSI/BSI-guidelines-2011.html
